# Biodiversity of bacteriophages: morphological and biological properties of a large group of phages isolated from urban sewage

**DOI:** 10.1038/srep34338

**Published:** 2016-10-04

**Authors:** Agata Jurczak-Kurek, Tomasz Gąsior, Bożena Nejman-Faleńczyk, Sylwia Bloch, Aleksandra Dydecka, Gracja Topka, Agnieszka Necel, Magdalena Jakubowska-Deredas, Magdalena Narajczyk, Malwina Richert, Agata Mieszkowska, Borys Wróbel, Grzegorz Węgrzyn, Alicja Węgrzyn

**Affiliations:** 1 Department of Molecular Evolution University of Gdańsk, Wita Stwosza 59, 80-308 Gdańsk, Poland; 2 Laboratory of Molecular Biology (affiliated with University of Gdańsk), Institute of Biochemistry and Biophysics, Polish Academy of Sciences, Wita Stwosza 59, 80-308 Gdańsk, Poland; 3 Department of Molecular Biology, and University of Gdańsk, Wita Stwosza 59, 80-308 Gdańsk, Poland; 4 Department of Genetics and Marine Biotechnology, Institute of Oceanology, Polish Academy of Sciences, Powstańców Warszawy 55, 81-712 Sopot, Poland; 5 Laboratory of Electron Microscopy, University of Gdańsk, Wita Stwosza 59, 80-308 Gdańsk, Poland; 6 Institute of Molecular Biology and Biotechnology, Adam Mickiewicz University in Poznań, Umultowska 89, 61-614, Poznań, Poland

## Abstract

A large scale analysis presented in this article focuses on biological and physiological variety of bacteriophages. A collection of 83 bacteriophages, isolated from urban sewage and able to propagate in cells of different bacterial hosts, has been obtained (60 infecting *Escherichia coli*, 10 infecting *Pseudomonas aeruginosa*, 4 infecting *Salmonella enterica*, 3 infecting *Staphylococcus sciuri*, and 6 infecting *Enterococcus faecalis*). High biological diversity of the collection is indicated by its characteristics, both morphological (electron microscopic analyses) and biological (host range, plaque size and morphology, growth at various temperatures, thermal inactivation, sensitivity to low and high pH, sensitivity to osmotic stress, survivability upon treatment with organic solvents and detergents), and further supported by hierarchical cluster analysis. By the end of the research no larger collection of phages from a single environmental source investigated by these means had been found. The finding was confirmed by whole genome analysis of 7 selected bacteriophages. Moreover, particular bacteriophages revealed unusual biological features, like the ability to form plaques at low temperature (4 °C), resist high temperature (62 °C or 95 °C) or survive in the presence of an organic solvents (ethanol, acetone, DMSO, chloroform) or detergent (SDS, CTAB, sarkosyl) making them potentially interesting in the context of biotechnological applications.

Bacteriophages were discovered about 100 years ago^1^. These viruses have played a tremendous role in the development of molecular biology and biotechnology^2^. To realize their importance in understanding molecular bases of biological processes, one can recollect that studies on bacteriophages led to the discovery (among other things) that DNA is a genetic material, that the genetic code is based on nucleotide triplets, and that gene expression is proceeded through mRNA molecules^3^,^4^. Bacteriophages have also played a crucial role in development of genetic engineering and biotechnology. In fact, first cloning vectors were based on bacteriophages, and commonly used systems for controlled gene expression and genetic recombination contain genes and regulatory sequences derived from bacteriophage genomes^5^. It is worth stressing that two breakthrough discoveries in biotechnology – identification of restriction enzymes and CRISPRs – are strictly connected to interaction mechanisms between bacteriophages and their hosts^6^. Nevertheless, until recent years, sufficient attention was not paid to the role of bacteriophages in natural environment or to biodiversity of these viruses. In fact, in molecular biology studies and biotechnological applications, only a very limited number of bacteriophages were employed, which was reasonable due to the choice of model organisms. However, it did not reflect the diversity of bacteriophages. Currently, this diversity appears extremely high. Bacteriophages are the most abundant form of life, as the number of phage entities on Earth is estimated at 10^31^, i.e. 10 times more than the number of bacterial cells^7^. In this light, our relatively poor knowledge of bacteriophage biodiversity is surprising. Although 1,910 complete bacteriophage genome sequences (and 77 genomes of viruses infecting archaea) have been deposited at the NCBI database, this number is low compared to 67,806 complete genome sequences of bacteria (as of 17^th^ April, 2016; http://www.ncbi.nlm.nih.gov/genome/). Nevertheless, from the available data one can deduce a tremendous diversity of bacteriophages, which is known only in a small fragment, as indicated in the newest papers focused on this problem^6^,^8^,^9^.

A vast majority of current studies on bacteriophage diversity focus on *in silico* analyses of nucleotide sequences of their genomes. Such studies provide extremely interesting information about the genetic variability of phage genomes, however, they also indicate that there is a large number of phage genes whose function cannot be predicted due to very low similarity, or lack thereof, to already known genes^9^. In this light, one may conclude that studies on newly isolated bacteriophages revealing interesting phenotypes should be of particular interest. Due to the tremendous biodiversity of bacteriophages, which is difficult to estimate in its entirety, G. F. Hatfull suggested that studies in this field may result in discoveries proving to be new milestones in biotechnology^6^. In fact, recent papers report the initiation of large-scale studies focused on discovering bacteriophages in environmental samples and analyses of their genomes^9^,^10^.

In our current project, we have applied a different strategy, which is a novel approach considering the previously published works on broad analyses of bacteriophages. While the large-scale genomic projects are based on isolation of large numbers of bacteriophages, sequencing of their genomes and advanced *in silico* analyses, in our studies, we focus on biodiversity of phages, i.e. investigating the biological and physiological variety of these viruses. Such analyses should indicate the level of biological diversity of bacteriophage properties in the sense of their development characteristics under various conditions. Apart from determining the level of variability of bacteriophages’ features, this could also provide the starting point for further studies on molecular mechanisms of putative phage uncommon traits. Moreover, this kind of research may provide a basis for determining newly discovered features of bacteriophages which can be useful in biotechnological and medical applications. Among them, construction of new vectors and development of bacteriophage therapy appear to be the most likely applicable novel aspects of such studies, as nowadays these fields of bacteriophage research are developing particularly rapidly^11^,^12^,^13^,^14^,^15^,^16^,^17^,^18^,^19^,^20^. In this work, particular focus has been put on morphological and biological characterization of a large group of bacteriophages isolated from urban sewage, which was assumed to be a rich source of these viruses. Such an innovative kind of study provided an example of the biodiversity level of bacteriophages isolated from a single place. Furthermore, potential biotechnological and medical applications of the results of these studies have emerged, rendering it possible to use these bacteriophages in constructing new tools for genetic engineering and bacteriophage therapy.

## Results and Discussion

### Isolation of bacteriophages

For isolation of bacteriophages from urban sewage, two strategies were applied. In one, two multiphage sewage samples were obtained by means of several steps of filtration of raw sewage samples, precipitation of viral-like particles (VLP), and their further purification. One hundred VLPs were selected at random from each of the two samples (called “multiphage samples 1 and 2”, or MPS1 and MPS2 respectively, further in the text) and subjected to morphological studies. In the other approach, 10 ml of a raw urban sewage sample was mixed with a culture of a particular bacterial strain, which was the first step to obtain lysates of bacteriophages able to propagate in cells of various hosts. A collection of 83 sewage phages (called “the collection” further in the text) infecting different bacterial strains has been created (Table 1). In this collection, there were 60 phages infecting *Escherichia coli* laboratory strains, 10 phages infecting *Pseudomonas aeruginosa* clinical strains, 4 phages infecting *Salmonella enterica* clinical strains, 3 phages infecting *Staphylococcus sciuri* strain (isolated from sewage), and 6 phages infecting *Enterococcus faecalis* strain (isolated from urban sewage) (Table 1). Coliphages were additionally investigated for their abilities to infect clinical isolates of *E. coli*. The results (Table 2), strongly suggest a possible use of these phages in further work on bacteriophage therapy, indicating that each tested enteropathogenic (EPEC) or enterohemorrhagic (EHEC) *E. coli* strain can be infected and lysed by at least one bacteriophage from the collection (in most cases by several or many phages). All phages from the collection were then subjected to morphological and physiological studies.

### Virion morphology

**Figure 1:**
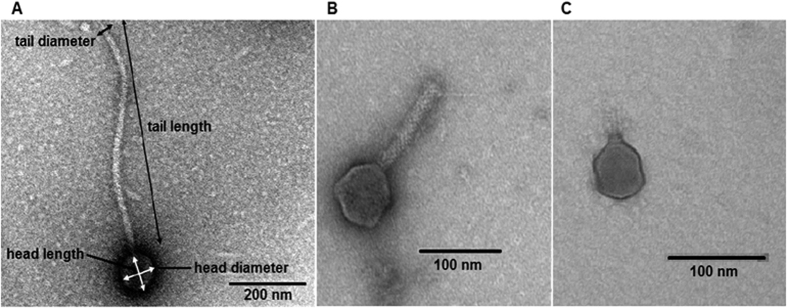
Examples of diversity of phage virions from the collection (**A** – *Siphoviride*, **B** –*Myoviridae*, **C** – *Podoviridae*). Indications for phage morphology parameters are shown in **A**.

Virion morphology of all phages isolated from the sewage (included in the collection, MPS1 and MPS2) was investigated by means of electron microscopic studies. All phages from the collection belonged to the order of tailed phages, *Caudovirales*. In multiphage samples (MPS1 and MPS2), we observed single filaments of morphological features resembling filamentous phages. However, knowing that identification of filamentous phages solely on the basis of microscopic analysis is difficult and prone to errors due to the risk of confusion with other filaments (like bacterial pili or flagella, and bacteriophage broken tails)^21^, we concentrated only on tailed viral particles. For their detailed classification, the following parameters were determined: head diameter (hd; width perpendicular to the tail), head length (along the tail axis; hl), tail diameter (td), and tail length (tl) (Fig. 1). Particles with tl < 40 nm were classified as *Podoviridae*, and the td value was used to classify viruses with longer tails as either *Myoviridae* (td ≥ 16 nm) or *Siphoviridae* (td < 16 nm), according to the previously published rule^22^.

Most of the phages, either from the collection or MPS1 and MPS2, belonged to the family *Siphoviridae*, characterized by long non-contractile tails. Most of them had isometric heads. There were some phages among *Sipho*- and *Myoviridae* with elongated heads with hl:hd ratio ≥ 1.3. Most myoviral particles had sheaths with noticeable characteristic criss-cross appearance, while some had visible side fibers. Phages from the family *Podoviridae*, characterized by short tails, were the least abundant (Table 3). When comparing physicochemical parameters of sewage samples used for isolation of bacteriophages (Table 4) with the percentage of isolated phages belonging to different families (Table 3), it appears that *Myoviridae* might exhibit higher survivability in poor environment conditions of their hosts than other families. MPS2 is significantly richer in *Myoviridae* than MPS1, and the corresponding sewage sample, MPS2, contained considerably lower amount of organic compounds, nitrogen, phosphorus and oxygen, relative to that of MPS1.

**Figure 2:**
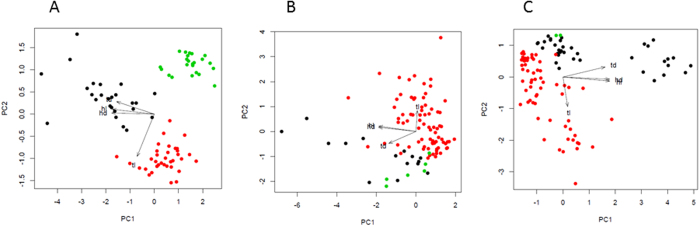
Principle component analysis for the collection (**A**), MPS1 (**B**), and MPS2 (**C**). Symbols: black, *Myoviridae*; red, *Siphoviridae*; green, *Podoviridae*; PC1, first principal component; PC2, second principle component; hl, head length; hd, head diameter; tl, tail length; td, tail diameter.

Principal component analysis was used to find any correlations between different parameters of phage particles. In order to check the statistical significance of the correlations, Spearman’s rank correlation coefficient (ϱ) and its statistical significance (*p*-value) were calculated. The data is presented in a biplot (Fig. 2), which is a graphical representation of the observations (points representing phages from the collection or MPS1 and MPS2) and the variables (vectors representing phage particles’ parameters). Points that are close to each other in the biplot correspond to the observations with similar values. The direction of the vectors has the highest squared multiple correlation with the principal components. The first principal component represents the direction of the maximum variation through the data – in our data it was the phage head diameter (hd), while the second principal component represents the second highest variation through the data – tail length (tl) (Fig. 2). The angle between vectors reflects the correlation between variables: the smaller the angle (0 < α < 90°) the higher the positive correlation between variables, and the higher the angle (90° < α < 180°), the higher the negative correlation between variables, while the right angle between the vectors indicates lack of correlation between variables.

Points representing phages from the collection in the biplot shown in Fig. 2A formed groups corresponding to three families of the order *Caudovirales*. Their head diameters were highly correlated with tail diameters (ϱ = 0.54, *p* = 1.24 × 10^−7^). Similarly, a high correlation was observed between head length and tail diameter (ϱ = 0.52, *p* = 3.76 × 10^−7^). Phages with tails of the largest diameter in the collection – myophages – had the largest capsids at the same time. Over a half of myophages in the collection with the average td = 20 nm had hd ≥ 70 nm, while 87% of podophages and 71% of siphophages with the average td = 11 nm, had hd < 60 nm (Supplementary Table S1).

High correlation between head size and tail diameter was also observed for the MPS2 (hd and td: ϱ = 0.57, *p* = 4.59 × 10^−10^; hl and td: ϱ = 0.60, *p* = 2.93 × 10^−11^) (Fig. 2C). More than a half of myophages with an average td of 19.6 nm had hd ≥ 70 nm (63% of them had hd > 100 nm). The correlation was less pronounced for MPS1 (hd and td: ϱ = 0.25, *p* = 0.01; hl and td: ϱ = 0.26, *p* = 0.01) (Fig. 2B). Here, only 33% of myophages with an average td: 19.6 nm, had hd ≥ 70 nm. Furthermore, in MPS1 and MPS2, we observed a larger fraction of siphophages with relatively big heads (hd ≥ 70 nm) than in the collection (10% in MPS1, 32% in MPS2 and only 6% in the collection, Supplementary Table S1).

Apart from the correlations described above, we have not observed any statistically significant correlations between tail length and the rest of parameters. Generally, phages with the longest tails (>200 nm) belonged to *Siphoviridae* (Supplementary Table S2). Interestingly, siphophages from MPS1 and MPS2 had longer tails than those from the collection in general (the longest one had tl = 598 nm) (Supplementary Table S2).

### Plaque morphology

**Figure 3:**
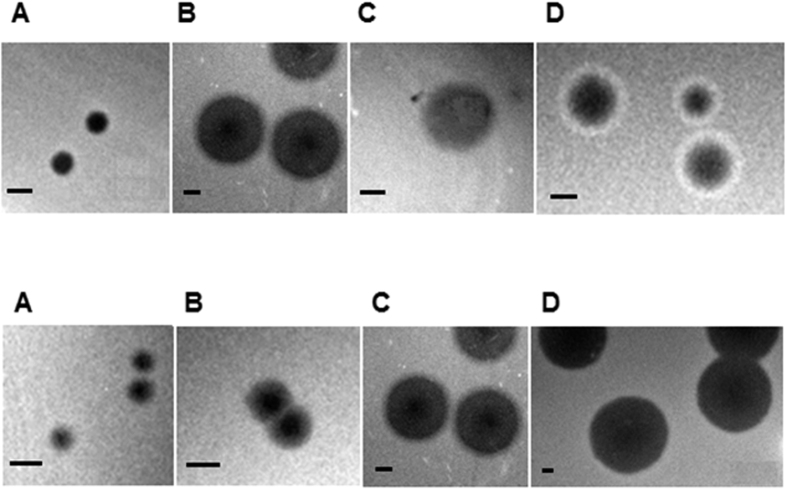
Examples of diversity of phage plaque morphology (upper panels) and plaque sizes (lower panels). In upper panels, following morphological types of plaques are shown: clear plaques (**A**), plaques with clear centers and turbid edges (**B**), turbid plaque (**C**), and plaques with a halo (**D).** In lower panels, plaques of different diameter (ϕ) are shown: ϕ < 1 mm (**A**), ϕ = 1.5 mm (**B**); ϕ = 4 mm (**C**), ϕ = 7 mm (**D**). The bar corresponds to 1 mm.

We characterized the turbidity and the diameter of plaques formed by bacteriophages from the collection (Table 1, Fig. 3). Most of the phages (74) formed clear plaques, typical for lytic (virulent) phages, while 9 phages formed turbid plaques which may indicate their ability to lysogenize host cells (temperate phages). Some plaques formed by coliphages displayed the “bull’s eye morphology”, i.e. greater turbidity toward plaque peripheries. Such morphology could be a consequence of decreasing lytic efficiency caused by aging of the bacterial lawn or the lysis inhibition phenomenon, observed previously among T-even phages^23^. For some phages, we observed haloes, i.e. semi-transparent zones around the plaques. The haloes could have stemmed from diffusion and the subsequent action of soluble (not virion associated), phage-produced enzymes destroying the cell envelope. One of the phages from the collection (vB_Pae575P-4), which produced plaques with a halo, was able to lyse the clinical, biofilm-forming *P. aeruginosa* strains, isolated from a patient with cystic fibrosis (Table 1).

Plaques also differed in size; the smallest ones had the diameter ≤ 1 mm, while the biggest ones about 7 mm. Principle component analysis revealed a correlation between head size (hd, hl) of the phages from the collection and plaque diameter (hd and pd: ϱ = −0.77, *p* < 2.2 × 10^−16^; hl and pd: ϱ = −0.75, *p* < 2.2 × 10^−16^). The phages with larger heads (large fraction of myophages) tend to form smaller plaques (Supplementary Table S3) in comparison to the phages with smaller heads (large fraction of sipho- and podophages). This was, presumably, due to the fact that larger virions (with larger heads) would diffuse more slowly through the top agar layer than smaller ones, thus resulting in smaller plaques. The ability of *Myoviridae* to form larger plaques than *Podoviridae* infecting the same host has previously been observed^24^. However, phages isolated by those authors differed morphologically only in tail length, having the same head size, suggesting that the latter parameter did not impact plaque size^24^.

### The host range

Phages from the collection showed a narrow host range, in most cases reduced to different strains of the same bacterial species (Table 1). Host range of 50 coliphages was the same as that of phage T4 for 3 out of 4 *E. coli* laboratory strains employed in this study (MG1655, TAP90, Hfr3000). Some coliphages were able, in addition, to infect clinical *E. coli* strains (Table 2). All phages isolated on *P. aeruginosa* caused the lysis of at least one clinical, biofilm-forming strain. All *E. faecalis* phages showed the ability to infect vancomycin-resistant enterococcal strains. Staphylococcal phages were able to lyse solely the strain they were isolated on. Meanwhile, two phages from the collection, isolated on *S. enterica* (vB_SenS-3 and vB_SenP-4), additionally caused the lysis of *E. coli* strains (Table 1). Bacteriophages are essentially host genus-specific in replication^25^. However, enterobacteria are related so closely that polyvalent phages are common among them, especially in the *E. coli*-*Shigella-Klebsiella* group^26^.

### Propagation temperature range

All phages from the collection were able to lyse their hosts at temperatures of 22 °C, 30 °C, and 37 °C (Table 1). The efficiency of plating was comparable for all three temperatures for all phages. Based on the effect of temperature on the efficiency of plating, three physiological types of bacteriophages were recognized previously^27^: high-temperature (HT) phages, plating at or above 25 °C, low-temperature (LT) phages, plating at or below 30 °C, and mid-temperature (MT) phages, plating in the range of 15–42 °C. *E. coli* phage T4, the archetype of the T4-type superfamily, is a typical representative of MT bacteriophages, as is the vast majority of the phages from the collection. Surprisingly, two myophages infecting *E. coli* (vB_Eco1M-13 and vB_Eco1M-21) showed the ability to lyse their hosts at low temperatures (4–7 °C) (Table 1). Similar results were described previously, when three low-temperature T4-like bacteriophages were isolated from Lithuanian municipal wastewater and sewage^27^. Formation of plaques at low temperatures is an unusual characteristic of coliphages. The inability of most bacteriophages to propagate under such conditions arises from blocks at various stages of phage development. For example, certain steps in T4 assembly seem to be inhibited at temperatures below 19 °C, which leads to accumulation of capsids, preheads, partially sheathed tails and naked cores^28^. In bacteriophage λ, DNA injection is ineffective at temperatures below 22 °C^29^. Therefore, efficient generation of progeny of phages vB_Eco1M-13 and vB_Eco1M-21 is particularly interesting. One might speculate that such phages could be of potential applicability when adaptation to grow at low temperatures is desirable, for example in wastewater treatment.

### Thermal inactivation

Bacteriophages were tested for survival at various temperatures, ranging from −20 °C to 95 °C. The vast majority of phages from the collection survived, to some extent, incubations at −20 °C and 40 °C, but they were sensitive to the temperature of 62 °C (Table 1). In most cases the phage titer dropped significantly after 40-min incubation at the latter temperature. However, three phages (vB_Eco1M-5, vB_Pae708M-2 and vB_Pae436M-8) showed high survivability (70–100%) at 62 °C; moreover phage vB_Eco4M-7 could survive even at 95 °C (Table 1). So far, the thermal inactivation of phages occurring in dewatered sludge and raw sewage has rarely been investigated. However, in such studies, significant reduction of the phage titer was observed after 30 min of incubation at 60 °C^30^. Based on the studies devoted to obtaining thermostable phage particles for biotechnological purposes, it was suggested that formation of disulfide cross-links within phage capsid proteins could play a role in stabilization of the phage against thermal denaturation^31^. Whether such stabilization occurs in vB_Eco1M-5, vB_Pae708M-2, vB_Pae436M-8 and vB_Eco4M-7 remains to be verified.

### Effects of the osmotic shock

**Figure 4:**
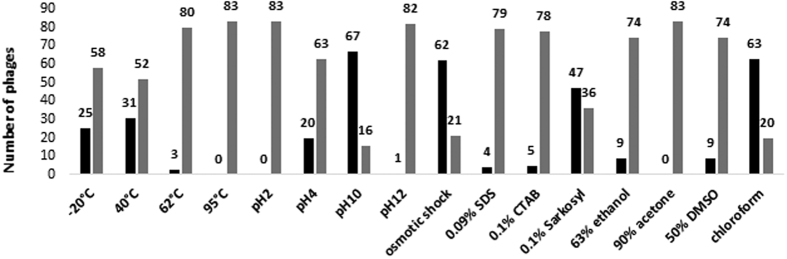
Comparison of effects of physical and chemical agents on phages from the collection. Black columns represent phages showing high survivability (70–100%), while grey columns indicate phages showing low survivability (below 70%).

The effects of the osmotic pressure change on phages from the collection caused by a rapid dilution of high-concentration salt solutions to low-concentration ones, were studied. Most of the phages (62 out of 83), showed high survivability (70–100%). Twenty-one phages were susceptible to osmotic shock (Table 1, Fig. 4). Interestingly, 18 of them belonged to *Myoviridae* and were distinguished by larger heads than the rest of phages from the collection (Table 1).

### The effect of high and low pH

The effects of high and low pH on phage virion stability were also studied. Almost all (80 out of 83) phages were stable at pH 10, and the titer of only 3 phages dropped by 80% or more after 1 h of incubation under these conditions (Table 1). On the other hand, most of the phages showed a significant decrease in the titer at pH 4 (Table 1, Fig. 4). However, 20 phages presented high survivability (70–100%) at this pH level (Table 1). None of the phages survived at pH 2, while quite a high fraction (48 out of 83) survived the incubation at pH 12 (Table 1).

### Effects of a detergent and organic solvents

The presence of detergents (0.09% SDS, 0.1% CTAB, 0.1% Sarkosyl) and organic solvents (63% ethanol, 90% acetone, 50% DMSO, chloroform) caused a significant titer drop of most phages from the collection, though the virions were quite resistant to chloroform. However, a few phages showed high survivability (70–100%) in the presence of SDS or ethanol (Table 1). One of them, vB_Eco1M-5, was also stable at 62 °C (Table 1), which indicates high resistance of the virion to various environmental conditions. Acetone was the factor with the highest anti-phage activity as all phages were more or less sensitive to this solvent (Fig. 4).

### Biodiversity of phages from the collection

To assess the level of biodiversity of bacteriophages from the collection, we compared all the phages in terms of morphology and physiology (the response to the studied conditions). We assumed that high biodiversity should allow us to construct a highly divergent dendrogram representing different levels of similarity between phages. However, such a dendrogram would be relatively simple if biodiversity of the studied phages was low. In the analysis, we considered virion morphology, plaque size, host spectrum, survivability at low and high temperatures, survivability at low and high pH, sensitivity to osmotic shock, sensitivity to a detergent (SDS) and organic solvents (see Table 1 for the considered features of each tested phage).

**Figure 5:**
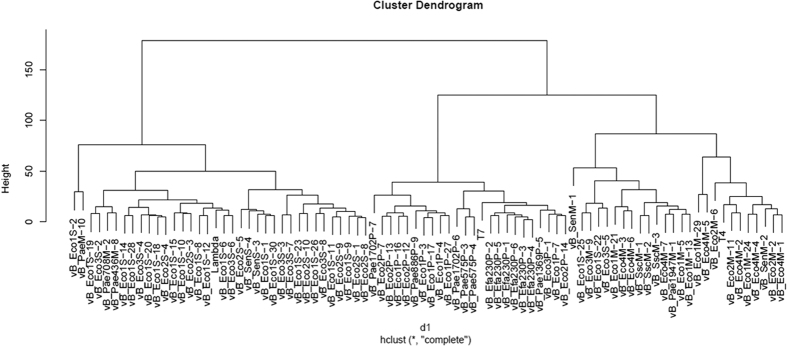
Dendrogram generated by hierarchical cluster analysis showing similarity/diversity between phages from the collection, and the level of similarity of the phages from the collection to reference phages (lambda, T7 and T4).

Using hierarchical cluster analysis, we generated a dendrogram which presented similarity/diversity among phages from the collection (Fig. 5). As external groups, reference phages λ (Siphoviridae), T7 (Podoviridae) and T4 (Myoviridae) were employed. The analysis showed high biodiversity among the tested bacteriophages, but also indicated that there were 32 pairs of similar phages. If each pair represented the same, single bacteriophage, then the number of isolated phages would be 51 rather than 83. To test this hypothesis, we compared phages from each pair. We considered phages to be highly similar when they met the following criteria: virions of high morphological similarity showed differences in head and tail size of no more than 10 nm, and phages of high physiological similarity had exactly the same host spectrum and belonged to the same or the adjacent sensitivity class. In this assessment, we distinguished four sensitivity classes: class I – phages showing 70–100% of survivability, class II - 40–69%, class III - 2–39%, class IV: 0–1%. Such a division considered possible experimental errors in calculating the relative phage titer. The performed verification reduced the number of phage pairs of high similarity because particular phages from several pairs differed in: host spectrum (vB_Eco1S-2 and vB_PaeM-10, vB_Pae708M-2 and vB_Pae436M-8, vB_Eco1P-16 and vB_Eco2P-12, vB_Eco4M-7 and vB_Pae1947M-1, vB_Eco1M-29 and vB_Eco4M-5, vB_Eco2M-11 and vB_Eco4M-2, vB_Eco1M-24 and vB_Eco4M-4, vB_Eco2M-2 and vB_Eco4M-1), sensitivity to thermal inactivation (vB_Eco1M-5 and vB_Eco1M-13), sensitivity to various pH (vB_Eco1S-1 and vB_Eco1S-30, vB_Eco3S-3 and vB_Eco3S-7, vB_Eco1S-26 and vB_Eco3S-8, vB_Eco1S-7 and vB_Eco2S-14), sensitivity to ethanol (vB_Eco1S-6 and vB_Eco3S-6, vB_Eco1S-11 and vB_Eco2S-9, vB_Eco1S-22 and vB_Eco3S-5) or sensitivity to DMSO (vB_Eco1S-18 and vB_Eco2S-4, vB_Eco1S-10 and vB_Eco2S-3, vB_Eco1S-25 and vB_Eco3M-9).

Assuming that the listed phages represent different viruses and that each remaining pair reflects one (the same) phage, different from other phages from the collection, the actual size of the collection would be 70 rather than 83 phages. This potential reduction, however, would not have a significant influence on phage distribution in families.

The results described here indicate high biodiversity of bacteriophages isolated from a single habitat. Previous reports also demonstrated diversity of phages infecting strains of Salmonella^8^ and Pseudomonas^32^. However, in those studies collections consisting of 55 and 22 phages, respectively, were employed (and called “large collections”), and the phages were tested using either lysis profiling, DNA restriction polymorphism, and random amplification of polymorphic DNA (for Salmonella phages)^8^ or virulence spectrum and DNA sequencing (for Pseudomonas phages)^32^. The report presented herein shows the comprehensible morphological and physiological analyses of at least 70 (and no more than 83) different bacteriophages isolated from a single environmental source, being perhaps the largest collection of phages investigated in such a way thus far.

### Genomic analyses

To investigate genetic variability among phages from the collection, complete genome sequences of bacteriophages vB_Efae230P-4, vB_Pae575P-3, vB_Pae1369P-5, vB_Pae436M-8, vB_SenM-2, vB_SscM-1, vB_SscM-2, have been determined and deposited in GenBank (accession numbers: JQ309827.1, KX171209, KX171210, KX171208, KX171211, KX171212, KX171213 respectively). Maps of these genomes are shown in Figs S1–S5. Annotations are described in Table S5.

Analyses of the sequenced genomes of selected phages indicated that they consist of linear double-stranded DNA of 17,972 bp (vB_Efae230P-4), 72,728 bp (vB_Pae575P-3), 72,508 bp (vB_Pae1369P-5), 66,922 bp (vB_Pae436M-8), 158,986 bp (vB_SenM-2), 139,681 bp (vB_SscM-1), and 139,682 bp (vB_SscM-2). The GC content was determined at 32.76%, 54.72%, 54.72%, 55.76%, 44.71%, 31.83% and 31.82%, respectively. Annotations of the sequenced genomes revealed 25, 92, 91, 91, 210, 203 and 202 coding DNA sequences (CDSs), respectively. Among all identified coding regions, relatively high percentages (56%, 72%, 76%, 60%,65%, 76% and 76%, respectively) of CDSs encoding hypothetical proteins have been determined. One might be surprised that uncharacterized coding sequences dominate in all analysed bacteriophage genomes, especially in the light of 24% of hypothetical proteins encoded in sequenced genomes of *E. coli* isolates (note also the size differences between genomes of analysed bacteriophages, ~0.1 Mb on average, and *E. coli* strains, ~4.6 Mb)^33^.

Genomic comparisons of the analysed phages to the most similar and previously sequenced phages from the NCBI database indicated that phage vB_Efae230P-4 presents 74% nucleotide sequence similarity with *Enterococcus* phage vB_IME195 (GenBank No. KT932700) whereas two other analysed phages: vB_Pae575P-3, and vB_Pae1369P-5 show 93% sequence similarity with *P. aeruginosa* phage PA26 (GenBank No. JX194238.1). All of them possess a *Podoviridae*-like morphology. The genomic sequence of phage vB_Pae436M-8 is similar in 95% to the other *P. aeruginosa* phage, named LAM2 (GenBank No. FM201282). Curiously, this phage belongs to *Myoviridae* family of long-tailed phages. Another analysed phage, vB_SenM-2, has also been classified into the *Myoviridae* family, but presents 94% sequence similarity with the *S. enterica* phage Det7 (GenBank No. KP797973.1). Two other analysed phages, vB_SscM-1 and vB_SscM-2, which have only 56% sequence similarity with *S. aureus* phage MCE-2014 (GenBank No. KJ888149.1) and no more closely related phage within NCBI database, have been classified as members of *Myoviridae* family too.

All the analysed phages share regions of greater or lesser level of sequence similarity with their reference phages. Although many coding sequences, identified within regions of high sequence identity (>50%), encode hypothetical proteins, there are also recognizable accessory genes with functions which were determined previously in other biological systems (Figs S1–S5). Curiously, despite a relatively high level of similarity to the reference phages’ sequences, all of the analysed phages possess significant regions of heterogeneity (sequence identity < 50%) which may influence their development. According to circular comparisons of phage genomes generated by using BLAST Ring Image Generator (BRIG), there are three such regions within the genome sequence of phage vB_Efae230P-4. The first fragment (from residue 3800 to 6163) includes two coding sequences predicted to encode putative calcineurin-like phosphoesterase, and a hypothetical protein. Interestingly, in the corresponding fragment of the *Enterococcus* phage vB_IME195 genome, two CDSs encoding proteins of unknown functions have been identified. The second analysed region within the vB_Efae230P-4 genome (from residue 16480 to16653) encompasses a 174-nt long CDS encoding a hypothetical protein. In a corresponding part of the vB_IME195 genome, there is a 881-nt long fragment with four CDSs predicted to encode uncharacterized proteins. The last analysedregion, extending from residue 17185 to 17972, of the vB_Efae230P-4 genome, shows a low level of sequence identity to vB_IME195 (<50%), however, similarly to the corresponding fragment of the vB_IME195 genome, it also includes two CDSs encoding hypothetical proteins (Supplementary Fig. S1).

According to the circular map shown in Supplementary Fig. S2, there is a fragment within genomic sequences of both vB_Pae575P-3 and vB_Pae1369P-5 (from residue 7044 to 7367) which includes one CDS (predicted to encode a hypothetical protein) and reveals low level of sequence identity (below 50%) when compared to *P. aeruginosa* phage PA26. An interesting region has also been identified within the genome sequence of phage vB_Pae436M-8 around residue 63100 (Fig. S3). In comparison with the DNA sequence of the reference phage LAM2, there is a deletion of a 268-nt-long fragment which in the case of phage LAM2 includes a CDS encoding a protein of an unknown function.

As indicated in the map shown in Fig. S4, in the genomic sequence of phage vB_SenM-2, there are several regions with low level of similarity to the reference phage Det7. Two of those regions, located in the vB_SenM-2 genome (at residues from 95873 to 96242, and from 131258 to 131794), arose from two independent insertions of DNA fragments with lengths of 369-nt and 536-nt, respectively. The first fragment includes a CDS which has no homology with any protein subjected to conventional functional analysis, whereas the other one encodes the Phage_tail_NK (PF16532) motif, which is characteristic for globular tip protein of some tailed bacteriophages^34^. Among the indicated differences between vB_SenM-2 and its reference phage Det7, there are also two deletions of 272-nt- and 134-nt-long fragments starting at residues 98152 and 115613 of the vB_SenM-2 genome sequence, respectively. Interestingly, with reference to the Det7 sequence, each of the deleted fragments includes one CDS encoding a hypothetical protein.

Unexpectedly, genome sequences of phages vB_SscM-1, vB_SscM-2, and MCE-2014 (found as the phage most similar to them) share only 56% sequence identity. As presented in Fig. S5, there are two large regions of low sequence identity (<50%) localized at both ends of vB_SscM-1 and vB_SscM-2 genomes. Significantly, the remaining parts of their genome sequences are also repeatedly interrupted by fragments sharing identity of below 50%. Such a mosaic structure allows to suppose that these phages may be results of productive recombination events. Intriguingly, vB_SscM-1 and vB_SscM-2 have been selected for sequencing from the analysed collection of phages because of the highest sensitivity to all analysed agents. The distinct behavior of these two phages and the fact that they are highly similar to each other (99% of sequence identity) and very different from other phages whose DNA sequences are available in NCBI database, suggest a qualification of vB_SscM-1 and vB_SscM-2 phages as a novel group of bacteriophages, and make them good candidates for further research.

In summary, our genomic comparisons of newly isolated phages from urban sewage to those published previously, reveal different levels of diversity and spotlight a continuous evolution in a single environmental source. The obtained data highlight the mosaicism of the analysed genomes, resulting from acquisition or loss of genetic material and numerous point mutations. Interestingly, sequencing data indicate that genomes of the analysed bacteriophages are littered with CDS predicted to encode homing endonucleases. They are site-specific DNA endonucleases that function as mobile genetic elements and are known as a significant source of genomic variability. The DNA repair process, catalyzed by these enzymes, results in transmission of the endonuclease gene and flanking segments of DNA between genomes^35^. It is remarkable to note that even a small sequence difference between the analysed and reference phages, when related to whole coding sequence, can make these newly isolated phages potentially interesting in the context of their biology. As a large portion of phage coding sequences are predicted to encode proteins of unknown functions, it is important to realize that the majority of viral diversity remains uncharacterized. In this light, the identification of genetic variations between phages and prediction of definitive or putative functions of hypothetical proteins are challenging and still remain a serious barrier to further progress in understanding the phage biology.

## Conclusions

Morphological and physiological analyses of a large collection of bacteriophages from a single environmental source indicated that there is high biodiversity among phages existing in one habitat. Some features of the investigated phages, like the ability to form plaques at low (4 °C) temperature, resistance to high (62 °C or even 95 °C) temperature or survival in the presence of organic solvents (ethanol, acetone, DMSO, chloroform) or detergents (SDS, CTAB, sarkosyl) make them potentially interesting in the context of biotechnological applications. Ability to propagate on clinical strains of bacteria suggest the potential use of these bacteriophages in phage therapy. Analysis of complete genome sequences of 7 from the newly isolated bacteriophages confirmed a high diversity of these viruses, even though they come from the single environmental source.

## Methods

### Samples for bacteriophage isolation, bacteria, and growth media

Raw sewage samples were collected at the Gdansk Wastewater Treatment Plant (Gdansk, Poland). The physicochemical parameters were provided by the Laboratory of Gdansk Wastewater Treatment Plant (Gdansk, Poland). Clinical strains of *Pseudomonas aeruginosa*, *Staphylococcus aureus* and *Salmonella enterica* were received from the National Medicines Institute in Warsaw (Poland), the Laboratory of Molecular Diagnostics of Intercollegiate Faculty of Biotechnology of University of Gdansk and the Medical University of Gdansk, and the National *Salmonella* Centre at Medical University of Gdansk (Poland), respectively. Enteropathogenic *E. coli* (EPEC) strains were isolated from stools of patients from the Specialist Hospital of St. Wojciech in Gdansk (Poland). Enterohemorrhagic *E. coli* (EHEC) strains, as well as laboratory strains of *E. coli* and *P. aeruginosa* were from the collection of the Department of Molecular Biology of the University of Gdansk (Poland). Environmental strains came from the collection of the Department of Water and Wastewater Technology of Gdansk University of Technology (*Enterococcus faecalis* strains isolated from urban sewage) and from the collection of the Institute of Oceanology of the Polish Academy of Sciences in Sopot (*Staphylococcus sciuri* strain isolated from urban sewage and *Pseudomonas* sp. isolated from marine sediments of the Baltic Sea). All bacterial strains employed in this study and detailed information about them are listed in the Supplementary Table S4.

For all phage experiments, *E. coli*, *Pseudomonas*, *Salmonella* and *Staphylococcus* bacteria were cultivated in Luria-Bertain (LB) liquid medium with aeration achieved by shaking, or plated on solid LB medium supplemented with 1.5% bacteriological agar. For enterococci special Todd Hewitt Broth (THB) and Brain-Heart-Infusion Agar (BHI agar) were prepared^36^.

### Double agar/agarose layer plates for phage propagation

The phage propagation procedure was performed according to the double overlay plaque assay with modifications^37^. For base plates preparation, standard Petri dishes were filled with 20–30 ml of LB or BHI medium containing 1.5% agar. In the next step, the 4 ml of the top LB or THB agar, supplemented with 0.4% agar/agarose, was mixed with 0.2 ml of the overnight bacterial cell culture and poured onto the bottom agar^38^. To determine the phage titer in a suspension (number of phages per ml), serial 10-fold dilutions were prepared in TM buffer (10 mM Tris–HCl, 10 mM MgSO_4_; pH 7.2) or culture media. Then, 2.5 μl of each dilution of the phage lysate were spotted on the double agar/agarose layer. Plates were incubated at 37 °C for 12–24 h and the phage concentration was quantified on the basis of the numbers of plaques.

### Isolation of bacteriophages

For bacteriophage isolation from sewage samples, two methods were employed. The first method was used for isolation of the whole phage population present in the sewage sample. Four liters of raw sewage were subjected to the elution procedure, as described earlier^39^, with some modifications. Briefly, every 0.5 l of raw sewage were mixed with 50 g of beef extract and agitated by magnetic stirring at room temperature for 30 min at 500–900 rpm. The extract was clarified by centrifugation (10,000× g, 30 min, 4 °C), and decontaminated by means of filtration through Millipore nitrocellulose filters (first with pore size 3 μm, then 0.45 μm) blocked with bovine serum albumin. VLPs were precipitated in the presence of 10% PEG 8,000 and 1 M NaCl (12 h at 4 °C with slow stirring, followed by centrifugation for 30 min, 10000× g). The precipitate was suspended in TM buffer (10 mM Tris-HCl, 10 mM MgSO_4_; pH 7.2) containing sodium chloride (final concentration 1 M) and extracted several times with chloroform to remove remnants of PEG and other contaminants. Multiphage lysates were kept at 4 °C for further analysis.

For the isolation of phages infecting particular bacterial strains one-host enrichment method was used, as described earlier^40^, with some modifications. Briefly, 10 ml of raw sewage were enriched with 1 ml of an overnight culture of a particular bacterial strain (one of 85 strains used), cultivated for 3–5 h at 37 °C with aeration achieved by shaking, and extracted several times with chloroform. One hundred microliters of each phage sample were added to an overnight culture of appropriate bacterial strain that was used in the enrichment step, and plated using the agar/agarose double layer method. Following incubation at 37 °C for 24 h, lysis zones were scraped, added to exponentially growing (in a liquid medium) host bacterial culture and cultivated at 37 °C until lysis occured. After chloroform extraction, phages were re-plated on a lawn of the strain from which they were originally isolated. Serial dilutions were prepared to obtain single phage plaques that were propagated three times by this method to ensure purity of the phage lysate. Isolated phages were named in accordance with the nomenclature of viruses of bacteria and archaea^41^.

### Electron microscopy

Purification of the virus-like particles (VLPs) was prepared using cesium chloride density gradient centrifugation procedure^42^. Electron microscopic analyses of the phage virions were performed employing the Philips CM 100 transmission electron microscope (Philips, Eindhoven, The Netherlands) operated at 80 kV, by using negative staining with uranyl acetate method, as described previously^43^. For phage identification, 2 μl of the investigated solution was absorbed onto carbon-coated 400 copper mesh grids, stained with 3% uranyl acetate (pH 4.5) for 15 s, and air-dried. Dimensions of virions were measured on micrographs at magnification of 39,000 times, with analySIS Pro (iTEM) software, and with a measuring magnifier, calibrated at 0.1 nm intervals.

### Plaque morphology analysis

Plaque morphology of all coliphages but one (vB_Eco4M-7 that lysed only *E. coli* O157:H7 ST2–8624) was tested on *E. coli* MG1655 strain. *P. aeruginosa* 436/1996 and *S. enterica* serovar Heidelberg were employed to analyse plaque morphology of *Pseudomonas* and *Salmonella* phages, respectively. To determine plaque size, serial ten-fold dilutions of phage stocks were prepared in LB or THB medium. In the next step, 0.2 ml of the host strain culture was mixed with 3 μl of an appropriate dilution of bacteriophage lysate and added to the 4 ml of the top LB or THB agar, supplemented with 0.4% agar/agarose. The mixture was poured onto an LB or BHI plate. The Petri dishes were incubated at 37 °C, and after 20 h, plaque morphology and diameter were assessed.

### Host range determination

To determine the host range of all isolated phages, different bacterial strains were tested (83 strains). The host range was created by observing the presence of plaques onto a double layer agar plate, prepared as described in the preceding paragraph. Ten-fold dilutions of phage stocks were performed in LB or THB medium, and spotted on the lawn of a potential host. Plates were incubated at 37 °C, and examined for plaques after 18–24 h.

### Propagation temperature range determination

To determine the propagation of bacteriophages at different temperatures, titers of the phage lysates were assayed after storage at 4 °C, 22 °C, 30 °C or 37 °C for 24 h, as described previously^27^. Ten-fold dilutions of phage stocks were prepared in LB or THB medium, and spotted on the lawn of the host strain, prepared by the double agar layer method. Efficiency of plating was assessed relative to results obtained at 37 °C.

### Thermal inactivation assessment

To estimate phage stability during thermal inactivation test, four different temperatures were investigated: −20 °C (12 h), 40 °C (40 min), 62 °C (40 min) and 95 °C (5 min). The procedure was conducted as described elsewhere^44^, with a minor modification. Phage lysate was diluted with LB or THB medium (at the volume proportion 1:9) and incubated under conditions described above. Next, the mixture was shortly withdrawn, serial 10-fold dilutions in appropriate medium were prepared and used for plating. Phages not subjected to thermal inactivation acted as a control. After overnight incubation at 37 °C, the percentage of remaining phages able to form plaques was calculated.

### The effect of various pH levels

The effect of an acidic and an alkaline pH on phage particles was studied using LB or THB medium with pH 2, pH 4, pH 10 and pH12, according to a procedure described previously^45^, with some modifications. Phage lysate were incubated for 1 h in the given medium (at the volume proportion 1:9) at 37 °C, and after preparation of serial 10-fold dilutions they were used for plating. To determine phage stability in various pH levels, phages incubated in the medium of pH 7 were used as a control. After 18 h of incubation in 37 °C, the percentage of phages able to lyse the host bacterial cells was estimated.

### The effect of osmotic shock

Measurement of survival of bacteriophages during osmotic shock conditions were studied by means of incubation of virus particles in TM buffer (10 mM Tris–HCl, 10 mM MgSO_4_; pH 7.2) containing sodium chloride (final concentration 4.5 M) at room temperature for 15 min^46^. Next, phage lysate was rapidly diluted in TM buffer without sodium chloride, and appropriate dilutions were dropped on double layer agar plates inoculated with appropriate host bacterial strain. Phages incubated in TM buffer without sodium chloride acted as a control.

### The effect of different detergents

To determine antiviral effect of ionic detergents on phage particles three different compounds were tested: anionic sodium dodecyl sulfate (SDS), anionic sodium lauroyl sarcosinate (Sarkosyl) and cationic cetyltrimethylammonium bromide (CTAB), according to the procedures described previously^47^,^48^,^49^, with some modification. Phage lysate was incubated with antimicrobial stock solution of 0.09% SDS (20 min at 45 °C), 0.1% Sarkosyl (10 min at 22 °C) and 0.1% CTB (1 min at 22 °C). The phage suspension was then diluted in TM buffer (10 mM Tris–HCl, 10 mM MgSO_4_; pH 7.2) and enumerated immediately by the double layer method. The control test was carried out identically without the addition of ionic detergents.

### The effect of organic solvents

The effect of four different organic solvents: ethanol, acetone, chloroform and dimethyl sulfoxide (DMSO) on phage virions, was studied. The procedures were conducted as described elsewhere^50^,^51^,^52^, with some modifications. A stock solution of phages was added to 63% ethanol, 90% acetone, chloroform and 50% DMSO. The mixtures were incubated for 1 h at 22 °C (ethanol and acetone), 1.5 h at 4 °C (chloroform) and 10 min at 4 °C (DMSO). In the next step, 10-fold dilutions in TM buffer (10 mM Tris–HCl, 10 mM MgSO_4_; pH 7.2) were prepared and used for plating. Phages incubated in TM buffer under conditions described above, were used as a control.

### Phage DNA isolation

The viral DNA was liberated from virions using the method described previously^53^, with a minor modification. To degrade the bacterial nucleic acids, all DNase- and RNase-treated samples were heated for 1 h at 37 °C. In the next step, the enzymes were inactivated for 10 min at 75 °C. The phage-encapsulated DNA of selected phages was then extracted with MasterPure Complete DNA Purification Kit according to the manufacturer’s recommendations. The DNA concentration was determined spectrophotometrically at 260 nm. DNA quality and size were controlled electrophoretically.

### Sequencing and analysis of phage genomes

Phage genomes were sequenced in the Max-Planck Institute (Cologne, Germany) using the pyrosequencing technology and Genome Sequencer FLX with Titanium series reagents. Assembly of the sequences was accomplished using MIRA^54^ and Consed^55^ programs. The sequencing results, in FASTA format, were analysed for any errors in contigs assembly using following programs: BLAST, available at the website: http://blast.ncbi.nlm.nih.gov/Blast.cgi, and Progressive MAUVE, available at: http://darlinglab.org/mauve/mauve.html. Any modifications within sequences were done using Serial Cloner, available at: http://serialbasics.free.fr/Serial_Cloner.html. Automatic annotations were carried out using myRAST software^56^, and later manually modified using standard text editor and UGENE bioinformatics software^57^, available at the website: http://ugene.net/. Sequence analysis and changes to the automatic annotations were done based on the data acquired by protein BLAST program (PSI-BLAST), HMMER software available at: http://www.ebi.ac.uk/Tools/hmmer/, Pfam database (http://pfam.xfam.org/), Phobious webserver (http://phobius.binf.ku.dk/), and TMHMM program available at: http://www.cbs.dtu.dk/services/TMHMM/. Putative coding sequences were added to the annotation list if they contained a plausible ribosome binding site, and both the start and stop codons. The.sqn files, which were sent to GenBank, were created using DNA Master software, available at: http://phagesdb.org/DNAMaster/. Circular comparisons between phage genomes and coordinates were generated using BLAST Ring Image Generator (BRIG) platform available at: https://sourceforge.net/projects/brig/.

### Statistical analysis

Principal Component Analysis (PCA) was used for the analysis of morphological and biological data. Spearman’s rank correlation coefficient (ϱ) and its statistical significance (*p* value) were calculated for the analysis of the correlations between variables. Correlations, for which p < 0.05 were considered statistically relevant. In addition, hierarchical clustering analysis, using Euclidean distance and complete linkage method, was used for statistical classification of data. All analyses were made employing R for Windows software, version 3.0.1.

### Statements on methodology

All methods were carried out in accordance with guidelines and regulations included in the official documents of University of Gdańsk. All experimental protocols were approved by the Research Committee of the Department of Molecular Biology of University of Gdańsk.

## Additional Information

**How to cite this article**: Jurczak-Kurek, A. *et al*. Biodiversity of bacteriophages: morphological and biological properties of a large group of phages isolated from urban sewage. *Sci. Rep*. **6**, 34338; doi: 10.1038/srep34338 (2016).

## Supplementary Material

Supplementary Information

Supplementary Data

## Figures and Tables

**Table 1:** Morphological and physiological characteristics of phages from the collection, and reference phages T4, λ, and T7.

Phage name	Phage family(hl, hd, tl, td)	Plaque morphology	Host spectrum*	Lysis at4 °C	Phage survivability in studied conditions (relative phage titer in %)															
					−20 °C(12 h)	40 °C(40 min)	62 °C(40 min)	95 °C(5 min)	pH 2 37 °C(1 h)	pH 4 37 °C(1 h)	pH 10 37 °C(1 h)	pH 12 37 °C(1 h)	Osmoticshock	0.09%SDS45 °C	0.1%CTAB(1 min)	0.1%Sarkosyl(10 min)	63%ethanol(1 h)	90%acetone(1 h)	50%DMSO(10 min)	chloro-form4 °C(1.5 h)
vB_Eco1S-1	*Siphoviridae* (65, 64, 167, 11)	clear; ø 1–1.7 mm	*E. coli* (MG1655, TAP90, Hfr3000)	−	77.5	77.5	48.9	0	0	68.6	100	9.9	100	0.9	12.3	100	10	2.4	6.4	41.4
vB_Eco1S-2	*Siphoviridae* (84, 76, 157, 10)	clear; ø 1–1.7 mm	*E. coli* (MG1655, TAP90, Hfr3000)	−	8.3	100	56	0	0	62.5	88.9	0	100	0.75	42.8	100	4.9	7.1	92.9	100
vB_Eco1P-3	*Podoviridae* (50, 48, 13, 13)	bull’s eye morphology; ø 2–6 mm	*E. coli* (MG1655, TAP90, Hfr3000)	−	58.3	50	0	0	0	10.6	88.2	0	100	0	0	100	0	0	0.04	40
vB_Eco1P-4	*Podoviridae* (56, 52, 9, 10)	bull’s eye morphology; ø 2–6 mm	*E. coli* (MG1655, TAP90, Hfr3000)	−	37.5	22.5	0	0	0	0	62.5	2.9	100	0	0	59	0	0	17.1	50
vB_Eco1M-5	*Myoviridae* (67, 67, 110, 22)	clear; ø 1–1.7 mm	*E. coli* (MG1655, TAP90, Hfr3000)	−	50	100	71.4	0	0	65	95	0.2	100	55.4	72.7	100	89.3	7.1	64.2	100
vB_Eco1S-6	*Siphoviridae* (54, 58, 147, 11)	clear; ø 2–3 mm	*E. coli* (MG1655, TAP90, Hfr3000)	−	45	45	11.8	0	0	4.8	79.3	1.7	100	0	24	100	100	17.4	100	90
vB_Eco1P-7	*Podoviridae* (46, 45, 29, 6)	clear; ø 3–5 mm	*E. coli* (MG1655, Tap90, Hfr3000)	+	59.1	41	0.6	0	0	3.2	100	0.02	100	0	0	100	8.3	0	0.04	13.3
vB_Eco1S-8	*Siphoviridae* (50, 48, 145, 11)	clear with halo; ø 3–5.5 mm	*E. coli* (MG1655, TAP90, Hfr3000)	−	83.3	35	30	0	0	9	84.6	4	100	0	0	100	40	14.4	92.9	100
vB_Eco1S-9	*Siphoviridae* (55, 54, 155, 10)	clear; ø 3–5 mm	*E. coli* (MG1655, TAP90, Hfr3000)	−	100	100	0.05	0	0	4	100	0.01	100	0	0	100	2	0.2	0.6	26.7
vB_Eco1S-10	*Siphoviridae* (61, 57, 135, 12)	bull’s eye morphology; ø 3–4 mm	*E. coli* (MG1655, TAP90, Hfr3000)	−	83.3	100	20	0	0	21	70	0.05	100	0	0.1	90	100	6.9	2.3	100
vB_Eco1S-11	*Siphoviridae* (62, 60, 158, 10)	bull’s eye morphology; ø 3–4 mm	*E. coli* (MG1655, TAP90, Hfr3000)	−	40	100	6.3	0	0	21.7	100	0.02	100	0	0	55	8.1	0.4	0	40
vB_Eco1S-12	*Siphoviridae* (49, 48, 141, 13)	bull’s eye morphology; ø 3–4 mm	*E. coli* (MG1655, TAP90, Hfr3000)	−	29	50	8.6	0	0	4.3	100	0	100	0	0.01	30.8	60.7	35.7	3.7	100
vB_Eco1M-13	*Myoviridae* (65, 63, 113, 18)	clear; ø 1–1.7 mm	*E. coli* (MG1655, TAP90, Hfr3000)	+	83.3	100	20.9	0	0	100	100	0	100	57	40	57.1	51.2	39	33.3	100
vB_Eco1S-14	*Siphoviridae* (48, 45, 136, 8)	clear with halo; ø 4–5 mm	*E. coli* (MG1655, TAP90, Hfr3000)	−	21.7	10	0.3	0	0	21.4	100	0	100	0	0	45.5	62.5	6.9	61.1	100
vB_Eco1S-15	*Siphoviridae* (61, 60, 141, 11)	clear; ø 2–3 mm	*E. coli* (MG1655, TAP90, Hfr3000)	−	15	75	1.2	0	0	1.3	90.3	0	100	0	0	37.3	1.4	17	45	60.4
vB_Eco1P-16	*Podoviridae* (47, 47, 9, 10)	clear; ø 5–7 mm	*E. coli* (MG1655, TAP90, Hfr3000)	−	41.7	33.3	0	0	0	6	100	2.7	100	0	0	90	0	0	0	100
vB_Eco1P-17	*Podoviridae* (52, 47, 11, 12)	clear; ø 5–7 mm	*E. coli* (MG1655, TAP90, Hfr3000)	−	33.3	66.7	0.4	0	0	10	100	0.01	100	0	0	100	0	0	12	100
vB_Eco1S-18	*Siphoviridae* (57, 51, 131, 10)	clear with halo; ø 4–6 mm	*E. coli* (MG1655, TAP90, Hfr3000)	−	100	66.7	0.01	0	0	21.2	100	0.1	88.9	0	0	70	2.8	0.01	2.7	100
vB_Eco1S-19	*Siphoviridae* (65, 63, 150, 10	bull’s eye morphology; ø 3–4.5 mm	*E. coli* (MG1655, TAP90, Hfr3000)	−	68.7	100	8.3	0	0	40	85.4	2.4	100	0	0	77.8	7.6	0.5	51.4	81.8
vB_Eco1S-20	*Siphoviridae* (57, 54, 132, 11)	clear with halo; ø 4–6 mm	*E. coli* (MG1655, TAP90, Hfr3000)	−	1	100	0	0	0	33.3	85.7	0	76.9	0	0.1	100	0.1	0.1	69.2	100
vB_Eco1M-21	*Myoviridae* (67, 73, 122, 21)	clear; ø 0.5–1 mm	*E. coli* (MG1655, TAP90, Hfr3000)	+	50	70	3	0	0	60	100	0.1	11	2.5	0	42.9	2.2	0.05	6.5	100
vB_Eco1S-22	*Siphoviridae* (49, 55, 106, 14)	turbid; ø 1–3 mm	*E. coli* (MG1655, TAP90, Hfr3000)	−	88.9	33.3	1.6	0	0	3	70	0.02	100	0.02	0.1	85.7	90	1.3	0.04	92.3
vB_Eco1S-23	*Siphoviridae* (51, 49, 155, 13)	clear; ø 3–5 mm	*E. coli* (MG1655, TAP90, Hfr3000)	−	83.3	100	23	0	0	12.5	100	0.1	100	0	0	66.7	70	9	2.9	100
vB_Eco1M-24	*Myoviridae* (86, 76, 102, 17)	clear; ø 0.5–1 mm	*E. coli* (MG1655, TAP90, Hfr3000)	−	7.3	8.2	1.8	0	0	33.3	80	16.6	7	24	0.04	19.2	0.5	0.01	0.3	63.2
vB_Eco1S-25	*Siphoviridae* (61, 59, 114, 10)	clear with halo; ø 3–5.5 mm	*E. coli* (MG1655, TAP90, Hfr3000)	−	35.7	28.6	17	0	0	79.2	100	10.9	100	0	0	100	60	4	0	100
vB_Eco1S-26	*Siphoviridae* (57, 58, 160, 13)	clear; ø 2–3 mm	*E. coli* (MG1655, Tap90, Hfr3000)	−	100	100	4.2	0	0	1.2	20.8	36.6	100	0.03	0.3	100	4.1	1.2	66.7	100
vB_Eco1P-27	*Podoviridae* (53, 51, 13, 9)	clear; ø 5–7 mm	*E. coli* (MG1655, TAP90, Hfr3000)	−	13.5	21.4	0	0	0	5	100	0.1	100	0	0.2	32.9	0	0	0	100
vB_Eco1S-28	*Siphoviridae* (50, 47, 132, 9)	clear with halo; ø 4–6 mm	*E. coli* (MG1655, TAP90, Hfr3000)	−	90	33.3	0.01	0	0	46.7	83.3	0	100	0	0.02	100	1.7	0	1.3	88.9
vB_Eco1M-29	*Myoviridae* (95, 88, 75, 21)	clear; ø 0.5–1 mm	*E. coli* (MG1655, TAP90, Hfr3000)	−	0.1	47.5	0.2	0	0	63.6	100	23.3	15	5.8	2.2	15	0.3	0.1	22	100
vB_Eco1S-30	*Siphoviridae* (65, 63, 171, 10)	clear with halo; ø 3–5.5 mm	*E. coli*(MG1655, TAP90, Hfr3000)	−	45	30	1.5	0	0	10	100	0.05	88.2	0	3.4	100	50	0.05	65.2	100
vB_Eco2S-1	*Siphoviridae* (58, 55, 157, 11)	bull’s eye morphology; ø 3–4 mm	*E. coli* (MG1655, TAP90, Hfr3000)	−	44.4	11.1	14.8	0	0	40.7	77.8	0.1	100	0	0	25.5	66.7	5.5	46.5	55.6
vB_Eco2M-2	*Myoviridae* (87, 68, 114, 16)	clear; ø 0.5–1 mm	*E. coli* (MG1655, TAP90, Hfr3000)	−	25	31.8	6.3	0	0	90	93.3	0	12	0	5	100	0	0	0	100
vB_Eco2S-3	*Siphoviridae* (56, 55, 140, 11)	clear with halo; ø 5–6 mm	*E. coli* (MG1655, TAP90, Hfr3000)	−	50	32	12.9	0	0	35.3	82.3	17.5	100	0.4	0.7	90	88.2	14.7	40	100
vB_Eco2S-4	*Siphoviridae* (54, 52, 130, 10)	bull’s eye morphology; ø 3–4 mm	*E. coli* (MG1655, TAP90, Hfr3000)	−	83.3	25	9.6	0	0	20.3	100	0.4	76.5	0	0	83.3	38.5	14.2	100	44.4
vB_Eco2S-5	*Siphoviridae* (71, 65, 173, 14)	clear; ø 0.5–1 mm	*E. coli* (MG1655, TAP90, Hfr3000)	−	46.6	31.7	21.7	0	0	33.3	56.7	1.3	100	9.7	0.4	4.5	0	0	15.7	100
vB_Eco2M-6	*Myoviridae* (104, 100, 103, 22)	clear; ø 0.5–1 mm	*E. coli* (MG1655, TAP90, Hfr3000)	−	18.2	14.2	4.2	0	0	81.8	100	0	1.3	3.5	8.9	18.2	0	0.03	0	100
vB_Eco2P-7	*Podoviridae* (46, 41, 13, 10)	clear; ø 5–7 mm	*E. coli* (MG1655, TAP90, Hfr3000)	−	10	20	0.03	0	0	1.4	100	0	100	0	0	100	0.2	0.2	0.02	100
vB_Eco2S-8	*Siphoviridae* (57, 56, 155, 10)	bull’s eye morphology; ø 2–3 mm	*E. coli* (MG1655, TAP90, Hfr3000)	−	66.7	21.7	12.5	0	0	12	80	30	88.9	0	1	100	100	50	66.7	100
vB_Eco2S-9	*Siphoviridae* (62, 59, 156, 10)	bull’s eye morphology; ø 2–3 mm	*E. coli* (MG1655, TAP90, Hfr3000)	−	57.1	17.4	17.5	0	0	7	100	2	100	0	0	75	77.3	10.9	43.3	100
vB_Eco2S-10	*Siphoviridae* (52, 47, 162, 11)	clear with halo; ø 3–5.5 mm	*E. coli* (MG1655, TAP90, Hfr3000)	−	50	20.5	18.9	0	0	11.1	72.2	10.6	100	0	0.3	27.3	72.2	50	59.3	80.9
vB_Eco2M-11	*Myoviridae* (96, 73, 109, 18)	clear; ø 1–1.7 mm	*E. coli* (MG1655, TAP90, Hfr3000)	−	62,5	19.4	0.2	0	0	100	89.6	0	1.3	0	1	100	0	0	0.01	100
vB_Eco2P-12	*Podoviridae* (44, 47, 9, 7)	bull’s eye morphology; ø 2–6 mm	*E. coli* (MG1655, TAP90)	−	16.7	22.2	0	0	0	0	20	6.7	100	0	0	100	0	0	0.02	8.2
vB_Eco2P-13	*Podoviridae* (46, 41, 10, 12)	bull’s eye morphology; ø 2–6 mm	*E. coli* (MG1655, TAP90, Hfr3000)	−	100	75	0.02	0	0	0.1	40.7	0.01	100	0	0	55	0	0	0.1	77.3
vB_Eco2P-14	*Podoviridae* (47, 48, 29, 10)	bull’s eye morphology; ø 2–6 mm	*E. coli* (MG1655, TAP90, Hfr3000)	−	100	100	0	0	0	0.2	15.8	0	100	0	0	100	0	0	0.2	28.3
vB_Eco3P-1	*Podoviridae* (50, 48, 28, 10)	bull’s eye morphology; ø 3–4 mm	*E. coli* (MG1655, Hfr3000)	−	64.3	4.8	0.6	0	0	25	100	0.1	100	0	0	47.2	38.3	21.7	100	100
vB_Eco3S-2	*Siphoviridae* (64, 57, 147, 11)	clear with halo; ø 4–6 mm	*E. coli* (MG1655, TAP90, Hfr3000)	−	0.01	96.2	0	0	0	47.6	95.2	39.4	100	0	0	0	0.01	0	66.7	71.4
vB_Eco3S-3	*Siphoviridae* (52, 48, 176, 11)	bull’s eye morphology; ø 3–4.5 mm	*E. coli* (MG1655, TAP90, Hfr3000)	−	31.3	25	6.5	0	0	22.8	100	2.5	92.7	0	0	38.5	4	0.3	38.9	100
vB_Eco3S-4	*Siphoviridae* (53, 54, 134, 9)	clear; ø 2–3 mm	*E. coli* (MG1655, TAP90, Hfr3000)	−	8.8	10	14.2	0	0	35	100	0.8	100	0	0	93.3	0.9	0.2	0.04	100
vB_Eco3S-5	*Siphoviridae* (47, 48, 116, 12)	bull’s eye morphology; ø 3–4.5 mm	*E. coli* (MG1655, TAP90, Hfr3000)	−	27.5	37.5	0	0	0	44.4	95.6	21.4	80	0	0	100	0	0	20	100
vB_Eco3S-6	*Siphoviridae* (52, 60, 144, 9)	bull’s eye morphology; ø 3–4 mm	*E. coli* (MG1655, TAP90, Hfr3000)	−	75	33.3	0.8	0	0	6.3	86.4	6.5	100	0	0	45	7.7	2.7	0	100
vB_Eco3S-7	*Siphoviridae* (56, 49, 179, 11)	clear; ø 1–2.5 mm	*E. coli* (MG1655, TAP90, Hfr3000)	−	6.3	7.9	0.04	0	0	75	34.3	0.1	100	0	0	100	0	0.6	12.7	100
vB_Eco3S-8	*Siphoviridae* (50, 59, 159, 13)	bull’s eye morphology; ø 2 mm	*E. coli* (MG1655, TAP90, Hfr3000)	−	31.7	41.7	0	0	0	50	0.1	0.04	100	0	0	100	0.01	9.4	12.2	100
vB_Eco3M-9	*Myoviridae* (61, 55, 114, 16)	bull’s eye morphology; ø 1–1.5 mm	*E. coli* (MG1655, TAP90, Hfr3000)	−	80	48	34.3	0	0	90.6	78.1	32	100	68.7	0	100	40.6	53.1	95.8	100
vB_Eco4M-1	*Myoviridae* (85, 68, 109, 18)	clear; ø 0.5–1 mm	*E. coli* (MG1655, TAP90, Hfr3000)	−	87.5	60	0	0	0	76.9	100	8	15	59.6	0	85.7	0	0	0	100
vB_Eco4M-2	*Myoviridae* (98, 76, 102, 18)	clear; ø 0.5–1 mm	*E. coli* (MG1655, TAP90, Hfr3000)	−	25	25	0	0	0	100	87.1	3.7	32.5	72.5	0	41.7	0	0	0	
vB_Eco4M-3	*Myoviridae* (80, 69, 124, 16)	clear; ø 0.5–1 mm	*E. coli* (MG1655, TAP90, Hfr3000)	−	10	100	0	0	0	91.5	93.6	0.3	20	62.7	0	100	0	0	0	100
vB_Eco4M-4	*Myoviridae* (82, 73, 99, 16)	clear; ø 0.5–1 mm	*E. coli* (MG1655, TAP90, Hfr3000)	−	35.7	100	0	0	0	100	100	0.1	14.1	50	0	40	0	0	0	75
vB_Eco4M-5	*Myoviridae* (98, 75, 52, 24)	clear; ø 0.5–1 mm	*E. coli* (MG1655, TAP90, Hfr3000)	−	65	100	0	0	0	89.5	84.2	0.6	23.6	65.4	0.2	100	0	0	0	79.4
vB_Eco4M-6	*Myoviridae* (76, 74, 115, 16)	clear; ø 0.5–1 mm	*E. coli* (MG1655, TAP90, Hfr3000)	−	33.3	47.5	0	0	0	100	88.9	1	19.5	65.8	0.3	66.7	0	0	0	77.8
vB_Eco4M-7	*Myoviridae* (71, 62, 104, 17)	turbid; ø 1 mm	*E. coli* (O157:H7 ST2–8624)	−	100	100	5.5	20.5	0	85	100	10	66.7	85.2	10	1.8	0	1.6	26.7	85.7
vB_Pae1947M-1	*Myoviridae* (66, 63, 102, 17)	turbid; ø <1 mm	*P. aeruginosa* (436/96, 1947/03, 1864/03)	−	77.8	25	15	0	0	57.1	64.3	65	100	42.4	69.6	100	11.5	0.03	24.7	100
vB_Pae708M-2	*Myoviridae* (67, 65, 141, 19)	clear; ø 1–2 mm	*P. aeruginosa* (436/96, 705/96, 708/96, 1369/03)	−	88.9	90	100	0	0.01	86.7	80	18.7	100	68.2	46.7	50	3.7	27.8	0.1	100
vB_Pae575P-3	*Podoviridae* (67, 63, 33, 10)	clear; ø 2 mm	*P. aeruginosa* (436/96, 705/96, 1947/03, 1864/03, 886/03, 1369/03, 575/03, 1702/09)	−	88.9	44.4	0	0	0	13.1	100	50	100	0	0	92.9	0	0	42.9	50
vB_Pae575P-4	*Podoviridae* (66, 65, 30, 10)	clear with halo; ø 2 mm	*P. aeruginosa* (436/96, 705/96, 1947/03, 1864/03, 886/03, 1369/03, 575/03, 1702/09)	−	22	100	0	0	0	10	100	73.3	100	0	0	50	0	0	15	56.7
vB_Pae1369P-5	*Podoviridae* (52, 52, 30, 9)	clear; ø 2 mm	*P. aeruginosa* (436/96, 705/96, 1947/03, 1864/03, 886/03, 1369/03, 575/03, 1702/09)	−	94.1	100	0	0	0	51.3	100	18.6	100	0	100	100	0	0	10.7	100
vB_Pae1702P-6	*Podoviridae* (67, 56, 39, 9)	clear; ø 2 mm	*P. aeruginosa* (436/96, 705/96, 1947/03, 1864/03, 886/03, 1369/03, 575/03, 1702/09)	−	100	100	0	0	0	40.8	100	17.5	70	0	0	78.3	0	0	43.7	66.7
vB_Pae1702P-7	*Podoviridae* (61, 61, 8, 10)	turbid; ø 3 mm	*P. aeruginosa* (436/96, 705/96, 1947/03, 1864/03, 886/03, 1369/03, 575/03, 1702/09)	−	100	100	0	0	0	53.5	100	34	100	0	100	100	0	0	100	100
vB_Pae436M-8	*Myoviridae* (60, 65, 146, 20)	clear; ø 1–2 mm	*P. aeruginosa* (436/96, 705/96, 708/96, 2317/03, 1369/03)	−	100	100	100	0	0	100	100	16.1	77.4	100	86.4	100	36.6	0.7	11.7	100
vB_Pae886P-9	*Podoviridae* (50, 54, 15, 14)	turbid; ø 2–3 mm	*P. aeruginosa* (436/96, 886/03)	−	43.9	81	0	0	0	26.3	84.7	12.9	94.4	1.2	1	77.8	0	0	9.2	100
vB_PaeM-10	*Myoviridae* (90, 91, 175, 26)	turbid ø<1 mm	*P. aeruginosa* (436/96, 708/96, 1000/03, 1947/03, 1864/03)	−	60	84	1.5	0	0	100	100	22.6	94.4	0	19	100	0	0	10	100
vB_SenM-1	*Myoviridae* (86, 35, 91, 16)	turbid; ø <1mm	*S. enterica* (Anatum, Heidelberg, London)	−	0	0.1	53.3	0	0	80	73.3	10	1.3	8.7	0	0.9	0	0	0	80
vB_SenM-2	*Myoviridae* (84, 79, 111, 18)	clear with halo; ø<1 mm	*S. enterica* (Anatum, Heidelberg, Panama)	−	1.5	0.02	56.2	0	0	100	56.2	0.01	37.5	100	3.9	18.8	0	0	0	77.8
vB_SenS-3	*Siphoviridae* (62, 61, 170, 11)	turbid; ø 1 mm	*S. enterica* (Heidelberg, Panama, Reading, London), *E. coli* (MG1655, TAP90, Hfr3000)	−	100	100	0.1	0	0	75	83.3	0.4	79.2	58.3	0	23.3	0.04	0	0	60
vB_SenS-4	*Siphoviridae* (64, 70, 166, 11)	turbid; ø <1 mm	*S. enterica* (Heidelberg, Panama, Reading), *E. coli* (MG1655, TAP90, Hfr3000)	−	43.8	58.3	0	0	0	10.5	51.2	2.8	87.8	0	0	64.9	0	0	15	100
vB_Efae230P-1	*Podoviriade* (48, 48, 18, 15)	clear with halo; ø 3–5 mm	*E. faecelis* (230, 423, 546)	−	16.3	0.2	0.2	0	0	15.5	100	0.4	81.8	12.7	4.8	12.9	0.1	0.02	0.01	10
vB_Efae230P-2	*Podoviriade* (50, 54, 22, 11)	clear with halo; ø 3–5 mm	*E. faecelis* (230, 423, 546)	−	9.1	0.2	0.1	0	0	10.8	100	2.5	83.3	15.8	17.2	88.2	0.1	0.1	0.1	52.4
vB_Efae230P-3	*Podoviridae* (52, 48, 22, 13)	clear with halo; ø 3–5 mm	*E. faecelis* (230, 423, 546)	−	21.8	0.05	0.1	0	0	13.8	50	18.3	28.7	15	30	58.3	0.2	0.03	0	100
vB_Efae230P-4	*Podoviridae* (52, 49, 21, 12)	clear with halo; ø 3–5 mm	*E. faecelis* (230, 423, 546)	−	5	0.01	0.1	0	0	11.5	69.2	15	69.2	8.5	24.1	66.7	0.1	0.01	0	60
vB_Efae230P-5	*Podoviridae* (45, 51, 22, 12)	clear with halo; ø 3–5 mm	*E. faecelis* (230, 423, 546)	−	27.8	0.5	1.9	0	0	11.7	100	34.6	75	12.5	4.8	37.5	0.1	0.02	0.7	76.7
vB_Efae230P-6	*Podoviridae* (49, 50, 22, 11)	clear with halo; ø 3–5 mm	*E. faecelis* (230, 423, 546)	−	19	0.8	0.2	0	0	16.4	81.8	30	36.4	12.7	50	10	0.1	0.01	0	30
vB_SscM-1	*Myoviridae* (72, 75, 109, 25)	clear; ø 1 mm	*S. sciuri* (IO)	−	0	100	0	0	0	0	17.5	0	0.03	0	0	76.5	0	0	100	100
vB_SscM-2	*Myoviridae* (74, 70, 107, 24)	bull’s eye morphology; ø 1 mm	*S. sciuri* (IO)	−	0	93.3	0	0	0	0	29.3	0.2	0	0	0	66.7	0	0	6.6	95.2
vB_SscM-3	*Myoviridae* (60, 62, 103, 25)	clear; ø 1 mm	*S. sciuri* (IO)	−	0	62.3	0	0	0	0	16.4	0	0.01	0	0	100	0	0	100	100
T4	*Myoviridae* (95, 65, 95, 21)	clear; ø 1–1.5	*E. coli* (MG1655, TAP90, Hfr3000)	−	40	40	28.6	0	0.02	100	100	3.2	9	4.4	75	75	0	0.2	0.03	85
λ	*Siphoviridae* (54, 54, 150, 15)	clear; ø 0.5–1	*E. coli* (MG1655, TAP90, Hfr3000)	−	4.2	15	0	0	0	32.3	100	57.5	100	0	0	74	0.03	0	60	72.4
T7	*Podoviridae* (55, 55, 29, 19)	bull’s eye morphology; ø 5–6	*E. coli* (MG1655, TAP90, Hfr3000)	−	1.5	100	0.04	0	0	35	100	6.4	100	0	0	42.5	0	0	23.9	100

*For the detailed information on all used bacterial host strains see the Supplementary Table S4. Abbreviations: (+) lysis after infection with tested bacteriophage, (−) a lack of lysis after infection with tested bacteriophage.

**Table 2:** Ability of coliphages from the collection to lyse the clinical strains of *E. coli*.

Phage name	Clinical strains of E. coli*					
	EPEC-A	EPEC-B	EPEC-C	O157:H7 ST2-8624	O157:H7 CB571	O157:H7 EDL933
vB_Eco1S-1	−	−	−	−	+	+
vB_Eco1S-2	−	−	−	−	+	+
vB_Eco1P-3	−	−	−	−	−	+
vB_Eco1P-4	−	−	−	−	−	+
vB_Eco1M-5	−	−	−	−	−	−
vB_Eco1S-6	−	−	+	−	+	−
vB_Eco1P-7	−	−	−	−	+	+
vB_Eco1S-8	−	−	−	−	+	+
vB_Eco1S-9	−	−	−	−	+	+
vB_Eco1S-10	−	−	−	−	+	+
vB_Eco1S-11	−	−	−	−	+	+
vB_Eco1S-12	−	−	−	−	+	+
vB_Eco1M-13	−	−	−	−	−	+
vB_Eco1S-14	−	−	−	−	+	+
vB_Eco1S-15	−	−	−	−	+	+
vB_Eco1P-16	−	−	−	−	−	+
vB_Eco1P-17	−	−	−	−	−	+
vB_Eco1S-18	−	−	−	−	−	+
vB_Eco1S-19	−	−	−	−	+	+
vB_Eco1S-20	−	−	−	−	+	+
vB_Eco1M-21	−	−	−	−	+	+
vB_Eco1S-22	−	−	−	−	+	+
vB_Eco1S-23	−	−	−	−	+	−
vB_Eco1M-24	−	+	+	−	+	−
vB_Eco1S-25	−	−	−	−	+	+
vB_Eco1S-26	−	−	−	−	+	+
vB_Eco1P-27	−	−	−	−	+	+
vB_Eco1S-28	−	−	−	−	+	+
vB_Eco1M-29	−	+	+	−	+	+
vB_Eco1S-30	−	−	−	−	+	+
vB_Eco2S-1	−	−	−	−	+	+
vB_Eco2M-2	+	+	+	−	+	+
vB_Eco2S-3	−	−	−	−	+	+
vB_Eco2S-4	−	−	−	−	+	+
vB_Eco2S-5	−	−	−	−	+	+
vB_Eco2M-6	−	+	+	+	+	+
vB_Eco2P-7	−	−	−	−	−	−
vB_Eco2S-8	−	−	+	−	+	+
vB_Eco2S-9	−	−	+	−	+	+
vB_Eco2S-10	−	−	−	−	+	+
vB_Eco2M-11	−	−	+	−	+	+
vB_Eco2P-12	−	−	−	−	−	−
vB_Eco2P-13	−	−	−	−	−	−
vB_Eco2P-14	−	−	−	−	−	−
vB_Eco3P-1	−	−	−	−	+	+
vB_Eco3S-2	−	−	−	−	+	+
vB_Eco3S-3	−	−	−	−	+	+
vB_Eco3S-4	−	−	−	−	+	+
vB_Eco3S-5	−	−	−	−	+	+
vB_Eco3S-6	−	−	−	−	+	+
vB_Eco3S-7	−	−	−	−	+	+
vB_Eco3S-8	−	−	−	−	+	+
vB_Eco3M-9	−	−	−	−	−	−
vB_Eco4M-1	−	−	+	+	+	+
vB_Eco4M-2	−	−	−	+	+	+
vB_Eco4M-3	−	−	−	+	−	+
vB_Eco4M-4	−	−	−	+	+	+
vB_Eco4M-5	−	−	−	+	+	+
vB_Eco4M-6	−	−	−	+	+	+
vB_Eco4M-7	−	−	−	+	−	−

*For the detailed information on *E. coli* clinical strains see the Supplementary Table S4. Abbreviations: (+) lysis after infection with tested bacteriophage, (−) a lack of lysis after infection with tested bacteriophage.

**Table 3:** Number of phages belonging to particular family in the collection and in MPS1 and MPS2.

Phages	Number of phages with particular morphology of the viral particle					
	*Myoviridae*		*Siphoviridae*		*Podoviridae*	
	Isometric heads	hl:hd ≥ 1.3	Isometric heads	hl:hd ≥ 1.3	Isometric heads	hl:hd ≥ 1.3
Collection	20	5	35	0	23	0
MPS1	15	0	76	3	6	0
MPS2	35	1	61	1	2	0

**Table 4:** Physicochemical parameters of sewage samples used for preparation MPS1 and MPS2.

Physicochemical parameter of sewage	Unit	Values of physicochemical parameters of sewage samples	
		MS1	MS2
General suspension	mg/l	468	343
Total dissolved solids (minerals)	mg/l	82	34
Volatile suspended solids	mg/l	386	309
pH	pH unit	7.00	7.19
Electrical conductivity	mS/cm	1.50	1.40
Sulfide	mg SO_4_^2−^/l	84	159
Chloride	mg Cl^−^/l	133	122
Total nitrogen	mg N/l	102	75
Ammonium nitrogen	mg N-NH_4_/l	69	54
Nitrate nitrogen	Mg N-NO_3_/l	1.06	0.50
Organic nitrogen	mg N_org_/l	32.11	20.58
Total phosphorus	mg P/l	14.00	9.05
Phosphate phosphorus	mg P-PO_4_/l	10.00	6.75
Biological oxygen demand	mg O_2_/l	608	327
Chemical oxygen demand	mg O_2_/l	878	615
