# Supplementary material for: Biodiversity of bacteriophages: morphological and biological properties of a large group of phages isolated from urban sewage
Source: Sci Rep. 2016 Oct 4;6:34338. doi: 10.1038/srep34338 (PMC5048108; doi:10.1038/srep34338)
Supplement: Supplementary Information [file srep34338-s1.pdf]

SUPPLEMENTARY MATERIAL

Biodiversity of bacteriophages: morphological and biological properties of a large group of phages isolated from urban sewage

Agata Jurczak-Kurek, Tomasz Gąsior, Bożena Nejman-Faleńczyk, Sylwia Bloch, Aleksandra Dydecka, Gracja Topka, Agnieszka Necel, Magdalena Jakubowska-Deredas, Magdalena Narajczyk, Malwina Richert, Agata Mieszkowska, Borys Wróbel, Grzegorz Węgrzyn, Alicja Węgrzyn

Table S1. The comparison of head diameter for phages of different families in the collection, MPS1 and MPS2

Phages	Phage family	Number of phages with head diameter:						
		30-40 nm	41-50 nm	51-60 nm	61-70 nm	71-100 nm	> 100 nm	Sum
Collection	*Myoviridae*	1	0	1	11	12	0	25
Collection	*Siphoviridae*	0	9	19	6	1	0	35
Collection	*Podoviridae*	0	13	7	3	0	0	23
MPS1	*Myoviridae*	0	4	4	2	3	2	15
MPS1	*Siphoviridae*	1	31	29	10	8	0	79
MPS1	*Podoviridae*	0	0	4	2	0	0	6
MPS2	*Myoviridae*	0	0	5	12	7	12	36
MPS2	*Siphoviridae*	0	1	29	14	17	1	62
MPS2	*Podoviridae*	0	0	0	0	2	0	2

Table S2. The comparison of tail length of phages from different families in the collection, MPS1 and MPS2

Phages	Phage family	Number of phages with tail length:					
		9-39 nm	50-100 nm	100-200 nm	200-300 nm	> 300 nm	Sum
Collection	*Myoviridae*	0	4	21	0	0	25
Collection	*Siphoviridae*	0	0	35	0	0	35
Collection	*Podoviridae*	23	0	0	0	0	23
MPS1	*Myoviridae*	0	3	12	0	0	15
MPS1	*Siphoviridae*	0	10	35	20	14	79
MPS1	*Podoviridae*	6	0	0	0	0	6
MPS2	*Myoviridae*	0	4	27	5	0	36
MPS2	*Siphoviridae*	0	2	24	20	16	62
MPS2	*Podoviridae*	2	0	0	0	0	2

Table S3. Plaque diameter of phages from the collection, belonging to different families

Plaque diameter (mm)	Number of phages		
	Myoviridae	*Siphoviridae*	*Podoviridae*
≤1	19	3	0
2	6	5	4
3-6	0	27	15
5-7	0	0	4

Table S4. Bacterial strains used in the study

Bacterial species/serovars	Source or reference	Isolation material or other characteristics	Antibiotic resistance
Pseudomonas aeruginosa 436/1996	National Medicines Institute in Warsaw (Poland)	patient with cystic fibrosis	gentamicin-resistant
Pseudomonas aeruginosa 705/1996	National Medicines Institute in Warsaw (Poland)	patient with cystic fibrosis	-
Pseudomonas aeruginosa 708/1996	National Medicines Institute in Warsaw (Poland)	patient with cystic fibrosis	gentamicin-resistant
Pseudomonas aeruginosa 954/1996	National Medicines Institute in Warsaw (Poland)	patient with cystic fibrosis	gentamicin and tobramycin-resistant
Pseudomonas aeruginosa 961/1996	National Medicines Institute in Warsaw (Poland)	patient with cystic fibrosis	gentamicin and tobramycin-resistant
Pseudomonas aeruginosa 781/2003	National Medicines Institute in Warsaw (Poland)	wound	gentamicin and tobramycin-resistant
Pseudomonas aeruginosa 1000/2003	National Medicines Institute in Warsaw (Poland)	wound	gentamicin and tobramycin-resistant
Pseudomonas aeruginosa 1947/2003	National Medicines Institute in Warsaw (Poland)	wound	gentamicin and tobramycin-resistant
Pseudomonas aeruginosa 2317/2003	National Medicines Institute in Warsaw (Poland)	wound	gentamicin and tobramycin-resistant
Pseudomonas aeruginosa 2838/2003	National Medicines Institute in Warsaw (Poland)	wound	gentamicin and tobramycin-resistant
Pseudomonas aeruginosa 2222/2003	National Medicines Institute in Warsaw (Poland)	decubitus ulcers	gentamicin and tobramycin-resistant
Pseudomonas aeruginosa 1864/2003	National Medicines Institute in Warsaw (Poland)	wound	-
Pseudomonas aeruginosa 1872/2003	National Medicines Institute in Warsaw (Poland)	post-operative wound	gentamicin and tobramycin-resistant
Pseudomonas aeruginosa 886/2003	National Medicines Institute in Warsaw (Poland)	post-operative wound	gentamicin-resistant
Pseudomonas aeruginosa 2652/2003	National Medicines Institute in Warsaw (Poland)	post-operative wound	gentamicin and tobramycin-resistant
Pseudomonas aeruginosa 1369/2003	National Medicines Institute in Warsaw (Poland)	wound	gentamicin and tobramycin-resistant
Pseudomonas aeruginosa 1900/2003	National Medicines Institute in Warsaw (Poland)	suppuration	gentamicin and tobramycin-resistant
Pseudomonas aeruginosa 2262/2003	National Medicines Institute in Warsaw (Poland)	post-operative wound	gentamicin and tobramycin-resistant

Pseudomonas aeruginosa 2734/2003	National Medicines Institute in Warsaw (Poland)	post-operative wound	gentamicin-resistant
Pseudomonas aeruginosa 575/2003	National Medicines Institute in Warsaw (Poland)	decubitus ulcers	gentamicin-resistant
Pseudomonas aeruginosa 1368/2003	National Medicines Institute in Warsaw (Poland)	decubitus ulcers	gentamicin-resistant
Pseudomonas aeruginosa 1702/2009	National Medicines Institute in Warsaw (Poland)	bronchial mucus	-
Pseudomonas aeruginosa KBM	Department of Molecular Biology of University of Gdansk (Poland)	wild type *P. aeruginosa*	-
Staphylococcus aureus 1893/2005	National Medicines Institute in Warsaw (Poland)	-	MRSA
Staphylococcus aureus 1391/2005	National Medicines Institute in Warsaw (Poland)	-	MRSA
Staphylococcus aureus 1932/2005	National Medicines Institute in Warsaw (Poland)	-	MRSA
Staphylococcus aureus 1781/2005	National Medicines Institute in Warsaw (Poland)	-	MRSA
Staphylococcus aureus 5069/2008	National Medicines Institute in Warsaw (Poland)	-	MRSA
Staphylococcus aureus 5074/2008	National Medicines Institute in Warsaw (Poland)	-	MSSA
Staphylococcus aureus 1881/2005	National Medicines Institute in Warsaw (Poland)	-	MSSA
Staphylococcus aureus 842/2005	National Medicines Institute in Warsaw (Poland)	-	MSSA
Staphylococcus aureus 421/2005	National Medicines Institute in Warsaw (Poland)	-	MSSA
Staphylococcus aureus 1088/2005	National Medicines Institute in Warsaw (Poland)	-	MSSA
Staphylococcus aureus 1899/1996	National Medicines Institute in Warsaw (Poland)	-	MSSA
Staphylococcus aureus 2977/2000	National Medicines Institute in Warsaw (Poland)	-	MSSA
Staphylococcus aureus 2233/2000	National Medicines Institute in Warsaw (Poland)	-	MSSA
Staphylococcus aureus 899/2003	National Medicines Institute in Warsaw (Poland)	-	MSSA
Staphylococcus aureus 303/2000	National Medicines Institute in Warsaw (Poland)	-	MSSA

Staphylococcus aureus 1365/2003	National Medicines Institute in Warsaw (Poland)	-	MSSA
Staphylococcus aureus 1562	Laboratory of Molecular Diagnostics of Intercollegiate Faculty of Biotechnology UG&MUG (Poland)	-	MRSA
Staphylococcus aureus 3659	Laboratory of Molecular Diagnostics of Intercollegiate Faculty of Biotechnology UG&MUG (Poland)	-	MRSA
Staphylococcus aureus 3578	Laboratory of Molecular Diagnostics of Intercollegiate Faculty of Biotechnology UG&MUG (Poland)	-	MRSA
Staphylococcus aureus 2002	Laboratory of Molecular Diagnostics of Intercollegiate Faculty of Biotechnology UG&MUG (Poland)	-	MRSA
Staphylococcus aureus 6504	Laboratory of Molecular Diagnostics of Intercollegiate Faculty of Biotechnology UG&MUG (Poland)	-	MRSA
Staphylococcus aureus 56/AS	Laboratory of Molecular Diagnostics of Intercollegiate Faculty of Biotechnology UG&MUG (Poland)	-	MRSA
Staphylococcus aureus 3442/1	Laboratory of Molecular Diagnostics of Intercollegiate Faculty of Biotechnology UG&MUG (Poland)	-	MRSA
Staphylococcus aureus 3442/2	Laboratory of Molecular Diagnostics of Intercollegiate Faculty of Biotechnology UG&MUG (Poland)	-	MRSA
Staphylococcus aureus 403/k/7	Laboratory of Molecular Diagnostics of Intercollegiate Faculty of Biotechnology UG&MUG (Poland)	community-aquired	MRSA
Staphylococcus aureus 264/k/7	Laboratory of Molecular Diagnostics of Intercollegiate Faculty of Biotechnology UG&MUG (Poland)	community-aquired	MRSA
Staphylococcus aureus 349/o/7	Laboratory of Molecular Diagnostics of Intercollegiate Faculty of Biotechnology UG&MUG (Poland)	community-aquired	MRSA
Staphylococcus aureus 345/o7	Laboratory of Molecular Diagnostics of Intercollegiate Faculty of Biotechnology UG&MUG (Poland)	community-aquired	MRSA
Staphylococcus aureus 201/s/7	Laboratory of Molecular Diagnostics of Intercollegiate Faculty of Biotechnology UG&MUG (Poland)	community-aquired	MRSA
Staphylococcus aureus 887/s/7	Laboratory of Molecular Diagnostics of Intercollegiate Faculty of Biotechnology UG&MUG (Poland)	community-aquired	MRSA
Staphylococcus aureus 127/0/s/7	Laboratory of Molecular Diagnostics of Intercollegiate Faculty of Biotechnology UG&MUG (Poland)	community-aquired	MRSA
Staphylococcus aureus 621/s	Laboratory of Molecular Diagnostics of Intercollegiate Faculty of Biotechnology UG&MUG (Poland)	community-aquired	MRSA
Staphylococcus aureus 2997/s	Laboratory of Molecular Diagnostics of Intercollegiate Faculty of Biotechnology UG&MUG (Poland)	community-aquired	MRSA
Staphylococcus aureus 7-43/s/7	Laboratory of Molecular Diagnostics of Intercollegiate Faculty of Biotechnology UG&MUG (Poland)	community-aquired	MRSA

Staphylococcus aureus 103/k/7	Laboratory of Molecular Diagnostics of Intercollegiate Faculty of Biotechnology UG&MUG (Poland)	community-aquired	MRSA
Staphylococcus sciuri IO	Institute of Oceanology of Polish Academy of Sciences in Sopot (Poland)	urban sewage isolate	
Salmonella enterica Anatum	National Salmonella Centre at Medical University of Gdansk (Poland)	-	
Salmonella enterica Heidelberg	National Salmonella Centre at Medical University of Gdansk (Poland)	-	
Salmonella enterica Panama	National Salmonella Centre at Medical University of Gdansk (Poland)	-	
Salmonella enterica Reading	National Salmonella Centre at Medical University of Gdansk (Poland)	-	
Salmonella enterica London	National Salmonella Centre at Medical University of Gdansk (Poland)	-	
Salmonella enterica Tennessee	National Salmonella Centre at Medical University of Gdansk (Poland)	-	
Enterococcus faecalis 227	Department of Water and Waste-Water Technology of Gdansk University of Technology	urban sewage isolate	VRE
Enterococcus faecalis 230	Department of Water and Waste-Water Technology of Gdansk University of Technology	urban sewage isolate	VRE
Enterococcus hirae 238	Department of Water and Waste-Water Technology of Gdansk University of Technology	urban sewage isolate	-
Enterococcus faecalis 405	Department of Water and Waste-Water Technology of Gdansk University of Technology	urban sewage isolate	-
Enterococcus faecalis 406	Department of Water and Waste-Water Technology of Gdansk University of Technology	urban sewage isolate	-
Enterococcus faecalis 423	Department of Water and Waste-Water Technology of Gdansk University of Technology	urban sewage isolate	VRE
Enterococcus faecium 450	Department of Water and Waste-Water Technology of Gdansk University of Technology	urban sewage isolate	-
Enterococcus faecalis 546	Department of Water and Waste-Water Technology of Gdansk University of Technology	urban sewage isolate	-
Enterococcus faecium 549	Department of Water and Waste-Water Technology of Gdansk University of Technology	urban sewage isolate	-
Escherichia coli MG1655 CGSC#6300	Blattner *et al*., 1997	-	-
Escherichia coli Tap90	Patterson & Dean, 1987	-	-

Escherichia coli Hfr3000	Bachmann, 1972	-	-
Escherichia coli EHEC O157:H7 ST2-8624	Griffin *et al*., 1988	stool; Stx1 & Stx2	-
Escherichia coli EHEC O157:H7 CB571	Beutin *et al*., 1989	stool ; Stx1 & Stx2	-
Escherichia coli EHEC O157:H7 EDL933	Beutin *et al*., 1989; Perna *et al*., 2001	stool; Stx1 & Stx2	-
Escherichia coli EPEC-A 129	Specialist Hospital of St. Wojciech in Gdansk (Poland)	stool ; EspA	-
Escherichia coli EPEC-B 21950	Specialist Hospital of St. Wojciech in Gdansk (Poland)	stool; EspB	-
Escherichia coli EPEC-C 22032	Specialist Hospital of St. Wojciech in Gdansk (Poland)	stool, EspC	-

MRSA – Methicillin-Resistant *S. aureus*, MSSA – Methicillin-Sensitive *S. aureus*, CA – Community-Aquired *S. aureus*, VRE – Vancomycin-resistant *Entercoccus*, EHEC – Enterohemorrhagic *E. coli*, EPEC – Enteropathogenic *E. coli*s, Stx – Shiga toxin, Esp – *E. coli* secretion protein

1. Blattner F. R., Plunkett G. 3rd, Bloch C. A., Perna N. T., Burland V., Riley M., Collado-Vides J., Glasner J. D., Rode C. K., Mayhew G. F., Gregor J., Davis N. W., Kirkpatrick H. A., Goeden M. A., Rose D. J., Mau B. & Shao Y. The complete genome sequence of *Escherichia coli* K-12. *Science* **277**, 1453-1462 (1997).
2. Patterson T. A., & Dean M. Preparation of high titer lambda phage lysates. *Nucleic Acids Res*. **15**, 6298 (1987).
3. Bachmann B. J. Pedigrees of some mutant strains of *Escherichia coli* K-12. *Bacteriol Rev*. **36**, 525-557 (1972).
4. Griffin, P. M., Ostroff, S. M., Tauxe, R. V., Greene, K. D., Wells, J. G., Lewis, J. H. & Blake P. A. Illnesses associated with *Escherichia coli* O157:H7 infections. A broad clinical spectrum. *Ann Intern Med*. **109**, 705-12 (1988).
5. Beutin, L., Montenegro, M. A. & Orskov, I. Close association of verotoxin (Shiga-like toxin) production with enterohemolysin production in strains of *Escherichia coli*. *J. Clin. Microbiol*. **27**, 2559–2564 (1989).
6. Perna, N. T., Plunkett, G., Burland, V., Mau, B., Glasner, J. D., Rose, D. J,. Mayhew, G. F., Evans, P. S., Gregor, J., Kirkpatrick, H. A., Pósfai, G., Hackett, J., Klink, S., Boutin, A., Shao, Y., Miller, L., Grotbeck, E. J., Davis, N. W., Lim, A., Dimalanta, E. T., Potamousis, K. D., Apodaca, J., Anantharaman, T. S., Lin, J., Yen, G. Schwartz, D. C., Welch, R. A. **&** Blattner, F. R. Genome sequence of enterohaemorrhagic *Escherichia coli* O157:H7. *Nature* **409**, 529-533 (2001).

Figures S1-S5. **BRIG-derived schematic maps of circular genomic comparisons between analyzed phages and reference phages selected from NCBI database as the most similar**. Image **S1** shows similarity between a central reference sequence of phage vB_IME195 (green ring) and sequence of the analyzed phage vB_Efae230P-4 (blue ring). Panel **S2** shows genomes of vB_Pae575P-3 (blue ring) and vB_Pae1369P-5 (red ring) compared against reference phage PA26 (green ring). Map **S3** presents comparison between vB_Pae436M-8 (blue ring) and the most similar phage LMA2 (green ring). Picture **S4** shows similarity between phage vB_SenM-2 (blue ring) and its reference phage Det7 (green ring). The last image **S5** presents genomic comparison between phages vB_SscM-1 (blue ring), vB_SscM-2 (red ring) and the most similar to them phage MCE-2014 (green ring). In the each map, BLAST matches are colored on a sliding scale indicating a defined percentage identity of compered sequences at three levels: 50%, 70% and 100%. The innermost rings show genome locations and GC content (black). The most external rings show results of genome annotation process of the each analyzed phage. The color of annotations corresponds to the color of the analyzed phage genome.

Figure S1

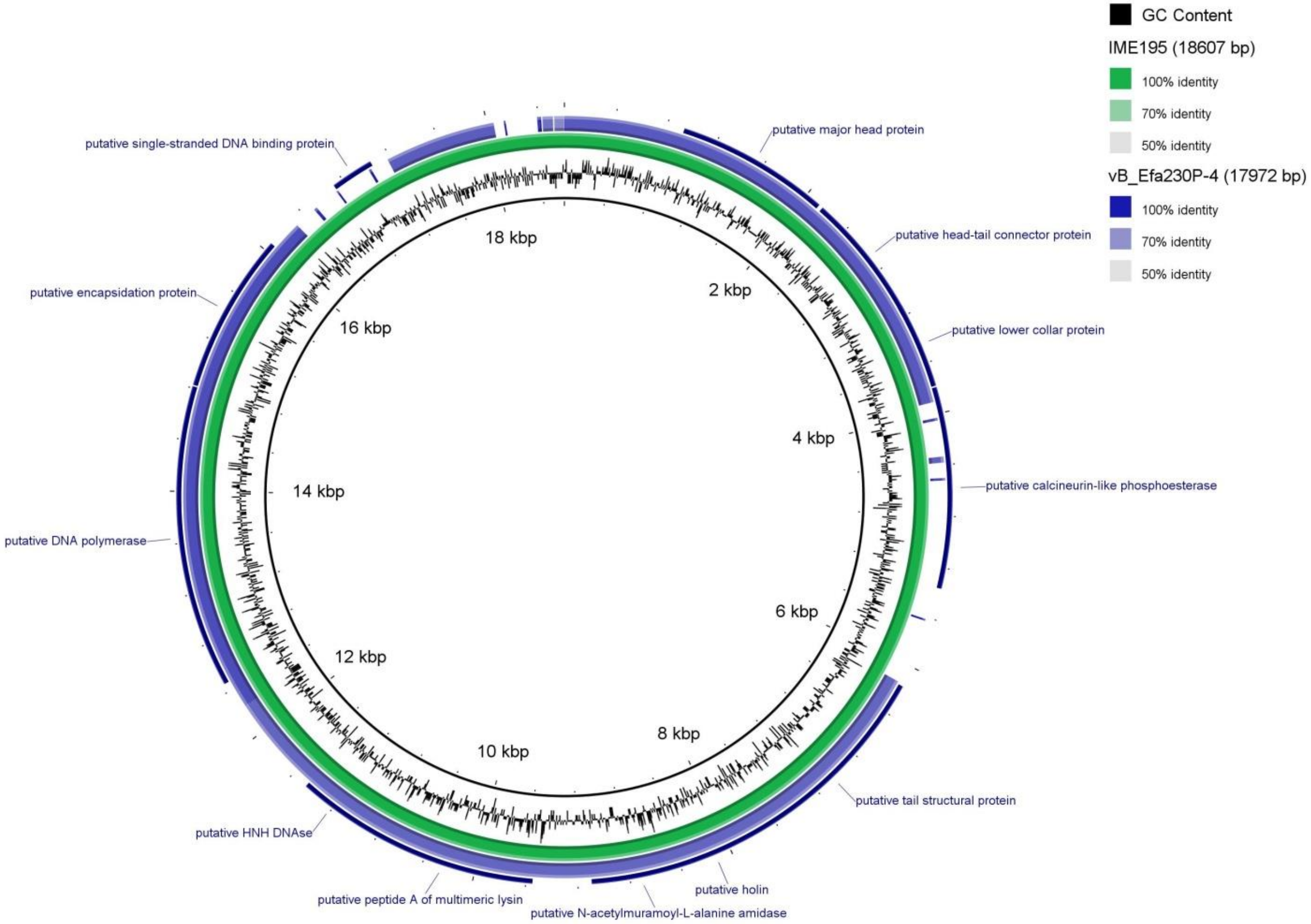
GC Content
IME195 (18607 bp)
100% identity
70% identity
50% identity
vB_Efa230P-4 (17972 bp)
100% identity
70% identity
50% identity
putative major head protein
putative head-tail connector protein
putative lower collar protein
putative calcineurin-like phosphoesterase
putative tail structural protein
putative holin
putative N-acetylmuramoyl-L-alanine amidase
putative peptide A of multimeric lysin
putative HNH DNAse
putative DNA polymerase
putative encapsidation protein
putative single-stranded DNA binding protein
2 kbp
4 kbp
6 kbp
8 kbp
10 kbp
12 kbp
14 kbp
16 kbp
18 kbp

Figure S2

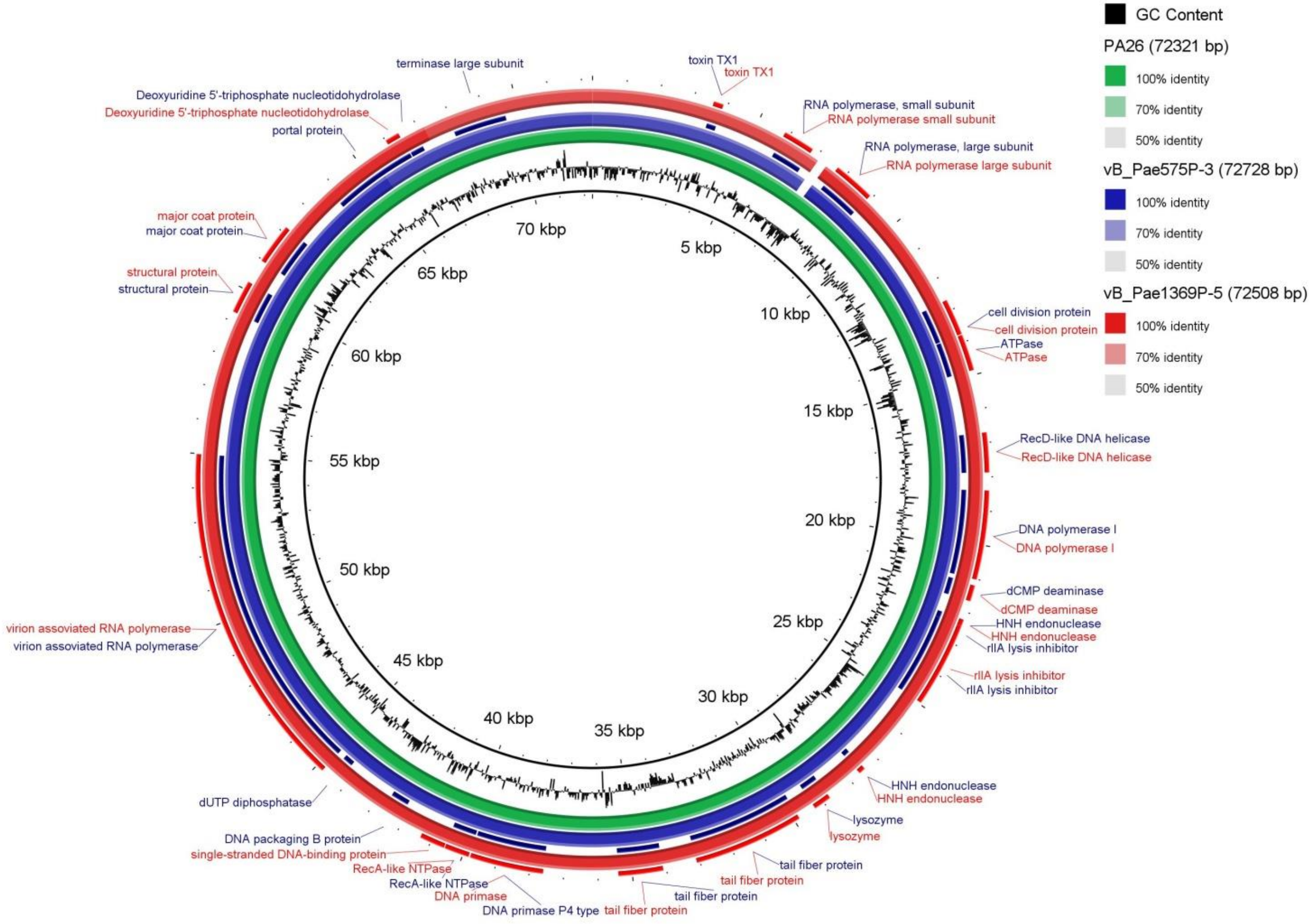

GC Content
PA26 (72321 bp)
100% identity
70% identity
50% identity
vB_Pae575P-3 (72728 bp)
100% identity
70% identity
50% identity
vB_Pae1369P-5 (72508 bp)
100% identity
70% identity
50% identity
terminase large subunit
toxin TX1
toxin TX1
Deoxyuridine 5'-triphosphate nucleotidohydrolase
Deoxyuridine 5'-triphosphate nucleotidohydrolase
portal protein
RNA polymerase, small subunit
RNA polymerase small subunit
RNA polymerase, large subunit
RNA polymerase large subunit
major coat protein
major coat protein
structural protein
structural protein
cell division protein
cell division protein
ATPase
ATPase
RecD-like DNA helicase
RecD-like DNA helicase
DNA polymerase I
DNA polymerase I
dCMP deaminase
dCMP deaminase
HNH endonuclease
HNH endonuclease
rIIA lysis inhibitor
rIIA lysis inhibitor
rIIA lysis inhibitor
virion assoviated RNA polymerase
virion assoviated RNA polymerase
HNH endonuclease
HNH endonuclease
lysozyme
lysozyme
dUTP diphosphatase
tail fiber protein
tail fiber protein
DNA packaging B protein
single-stranded DNA-binding protein
RecA-like NTPase
RecA-like NTPase
DNA primase
DNA primase P4 type
tail fiber protein
tail fiber protein
70 kbp
5 kbp
65 kbp
10 kbp
60 kbp
15 kbp
55 kbp
20 kbp
50 kbp
25 kbp
45 kbp
30 kbp
40 kbp
35 kbp

Figure S3

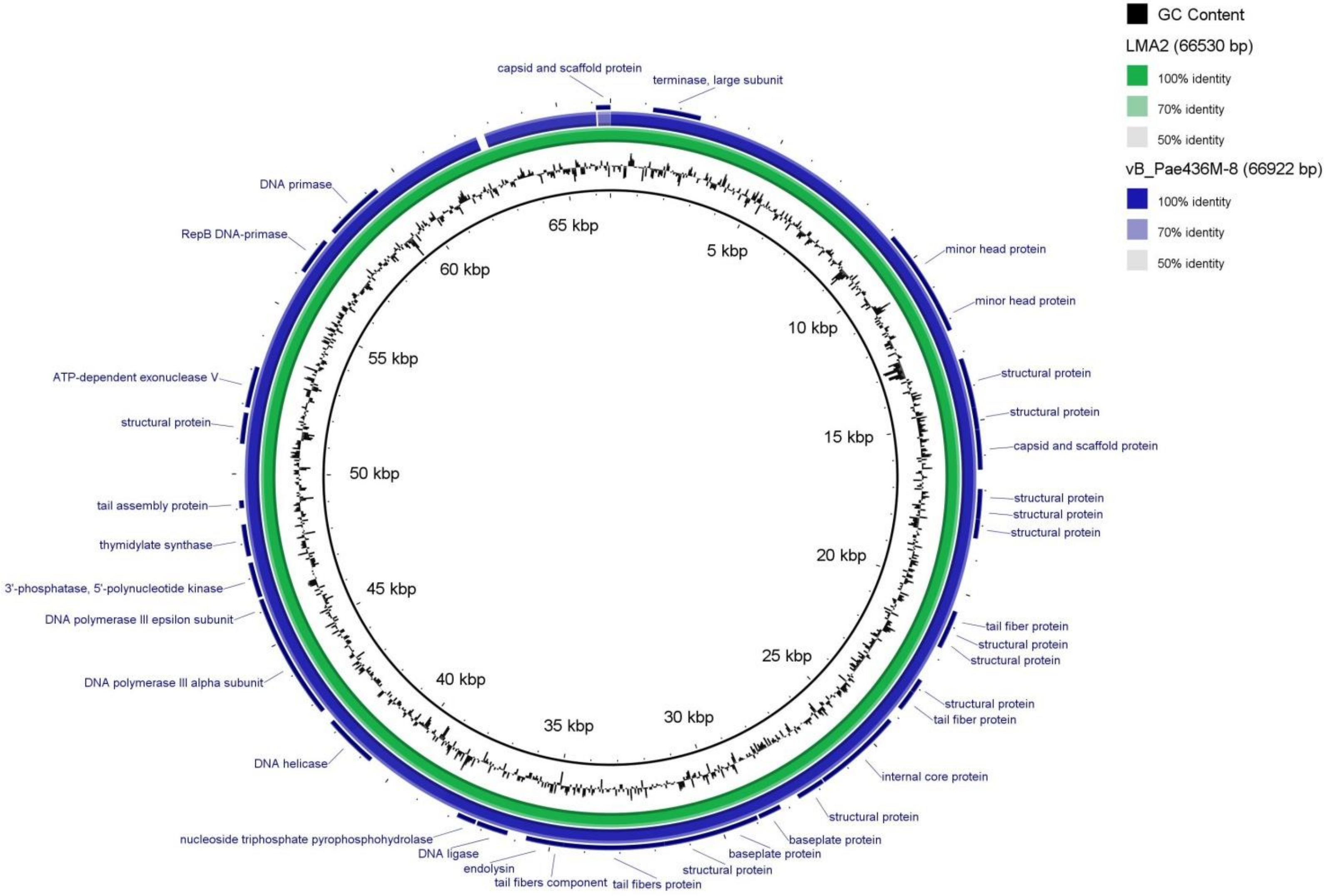
GC Content
LMA2 (66530 bp)
100% identity
70% identity
50% identity
vB_Pae436M-8 (66922 bp)
100% identity
70% identity
50% identity
capsid and scaffold protein
terminase, large subunit
minor head protein
minor head protein
structural protein
structural protein
capsid and scaffold protein
structural protein
structural protein
structural protein
tail fiber protein
structural protein
structural protein
structural protein
tail fiber protein
internal core protein
structural protein
baseplate protein
baseplate protein
structural protein
tail fibers protein
tail fibers component
endolysin
DNA ligase
nucleoside triphosphate pyrophosphohydrolase
DNA helicase
DNA polymerase III alpha subunit
DNA polymerase III epsilon subunit
3'-phosphatase, 5'-polynucleotide kinase
thymidylate synthase
tail assembly protein
structural protein
ATP-dependent exonuclease V
RepB DNA-primase
DNA primase
5 kbp
10 kbp
15 kbp
20 kbp
25 kbp
30 kbp
35 kbp
40 kbp
45 kbp
50 kbp
55 kbp
60 kbp
65 kbp

Figure S4

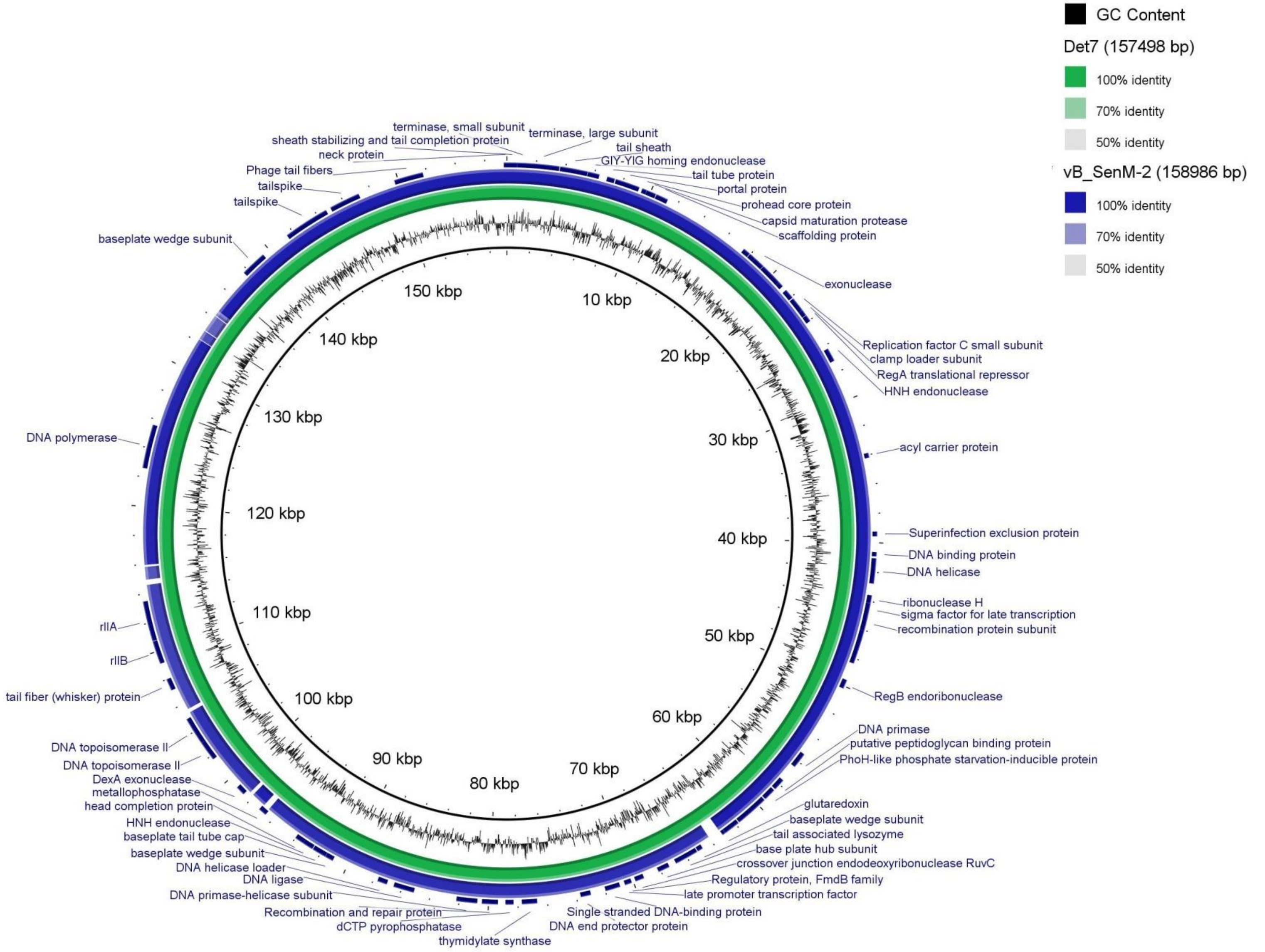
GC Content
Det7 (157498 bp)
100% identity
70% identity
50% identity
vB_SenM-2 (158986 bp)
100% identity
70% identity
50% identity
10 kbp
20 kbp
30 kbp
40 kbp
50 kbp
60 kbp
70 kbp
80 kbp
90 kbp
100 kbp
110 kbp
120 kbp
130 kbp
140 kbp
150 kbp
terminase, small subunit
terminase, large subunit
sheath stabilizing and tail completion protein
tail sheath
neck protein
GIY-YIG homing endonuclease
Phage tail fibers
tail tube protein
tailspike
portal protein
tailspike
prohead core protein
capsid maturation protease
scaffolding protein
baseplate wedge subunit
exonuclease
Replication factor C small subunit
clamp loader subunit
RegA translational repressor
HNH endonuclease
DNA polymerase
acyl carrier protein
Superinfection exclusion protein
DNA binding protein
DNA helicase
ribonuclease H
sigma factor for late transcription
recombination protein subunit
rIIA
rIIB
tail fiber (whisker) protein
RegB endoribonuclease
DNA primase
putative peptidoglycan binding protein
PhoH-like phosphate starvation-inducible protein
DNA topoisomerase II
DNA topoisomerase II
DexA exonuclease
metallophosphatase
head completion protein
HNH endonuclease
baseplate tail tube cap
baseplate wedge subunit
DNA helicase loader
DNA ligase
DNA primase-helicase subunit
glutaredoxin
baseplate wedge subunit
tail associated lysozyme
base plate hub subunit
crossover junction endodeoxyribonuclease RuvC
Regulatory protein, FmdB family
late promoter transcription factor
Single stranded DNA-binding protein
DNA end protector protein
Recombination and repair protein
dCTP pyrophosphatase
thymidylate synthase

Figure S5

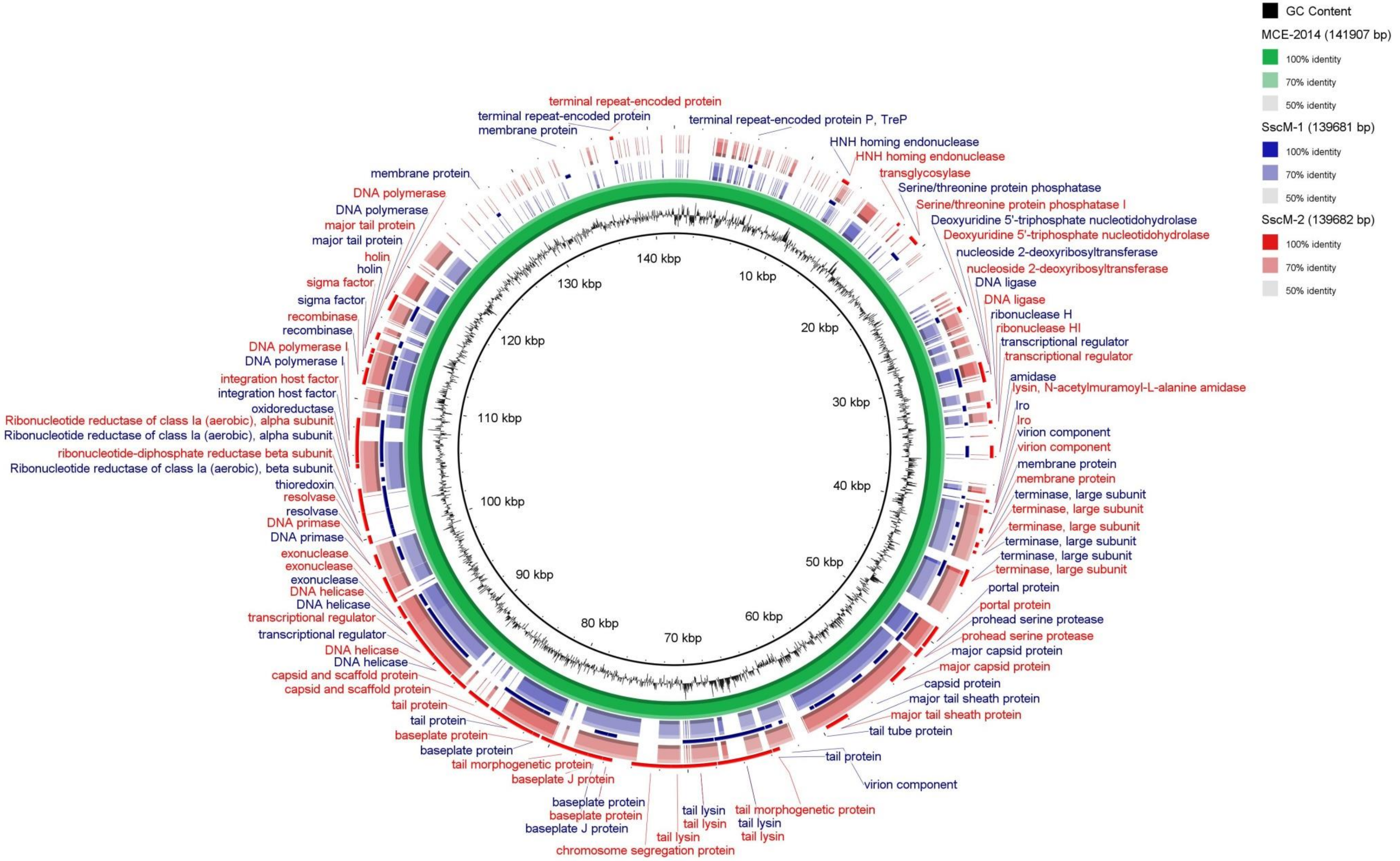
GC Content
MCE-2014 (141907 bp)
100% identity
70% identity
50% identity
SscM-1 (139681 bp)
100% identity
70% identity
50% identity
SscM-2 (139682 bp)
100% identity
70% identity
50% identity
10 kbp
20 kbp
30 kbp
40 kbp
50 kbp
60 kbp
70 kbp
80 kbp
90 kbp
100 kbp
110 kbp
120 kbp
130 kbp
140 kbp
terminal repeat-encoded protein
terminal repeat-encoded protein
membrane protein
terminal repeat-encoded protein P, TreP
HNH homing endonuclease
HNH homing endonuclease
transglycosylase
Serine/threonine protein phosphatase
Serine/threonine protein phosphatase I
Deoxyuridine 5'-triphosphate nucleotidohydrolase
Deoxyuridine 5'-triphosphate nucleotidohydrolase
nucleoside 2-deoxyribosyltransferase
nucleoside 2-deoxyribosyltransferase
DNA ligase
DNA ligase
ribonuclease H
ribonuclease HI
transcriptional regulator
transcriptional regulator
amidase
lysin, N-acetylmuramoyl-L-alanine amidase
lro
lro
virion component
virion component
membrane protein
membrane protein
terminase, large subunit
terminase, large subunit
terminase, large subunit
terminase, large subunit
terminase, large subunit
terminase, large subunit
portal protein
portal protein
prohead serine protease
prohead serine protease
major capsid protein
major capsid protein
capsid protein
major tail sheath protein
major tail sheath protein
tail tube protein
tail protein
virion component
tail morphogenetic protein
tail lysin
tail lysin
tail lysin
tail lysin
tail lysin
chromosome segregation protein
baseplate protein
baseplate protein
baseplate J protein
baseplate J protein
tail morphogenetic protein
baseplate protein
baseplate protein
tail protein
tail protein
capsid and scaffold protein
capsid and scaffold protein
DNA helicase
DNA helicase
transcriptional regulator
transcriptional regulator
DNA helicase
DNA helicase
exonuclease
exonuclease
exonuclease
DNA primase
DNA primase
resolvase
resolvase
thioredoxin
Ribonucleotide reductase of class Ia (aerobic), beta subunit
ribonucleotide-diphosphate reductase beta subunit
Ribonucleotide reductase of class Ia (aerobic), alpha subunit
Ribonucleotide reductase of class Ia (aerobic), alpha subunit
oxidoreductase
integration host factor
integration host factor
DNA polymerase I
DNA polymerase I
recombinase
recombinase
sigma factor
sigma factor
holin
holin
major tail protein
major tail protein
DNA polymerase
DNA polymerase
membrane protein
